# The internal cranial anatomy of *Champsosaurus* (Choristodera: Champsosauridae): Implications for neurosensory function

**DOI:** 10.1038/s41598-020-63956-y

**Published:** 2020-04-28

**Authors:** Thomas W. Dudgeon, Hillary C. Maddin, David C. Evans, Jordan C. Mallon

**Affiliations:** 1 Department of Earth Sciences, Carleton University, Ottawa, Canada; 2 Vertebrate Palaeontology, Royal Ontario Museum, Toronto, Canada; 3 Department of Ecology and Evolutionary Biology, University of Toronto, Toronto, Canada; 4 Beaty Centre for Species Discovery and Palaeobiology Section, Canadian Museum of Nature, Ottawa, Canada

**Keywords:** Ecology, Evolution, Zoology

## Abstract

Although isolated *Champsosaurus* remains are common in Upper Cretaceous sediments of North America, the braincase of these animals is enigmatic due to the fragility of their skulls. Here, two well-preserved specimens of *Champsosaurus* (CMN 8920 and CMN 8919) are CT scanned to describe their neurosensory structures and infer sensory capability. The anterior portion of the braincase was poorly ossified and thus does not permit visualization of a complete endocast; however, impressions of the olfactory stalks indicate that they were elongate and likely facilitated good olfaction. The posterior portion of the braincase is ossified and morphologically similar to that of other extinct diapsids. The absence of an otic notch and an expansion of the pars inferior of the inner ear suggests *Champsosaurus* was limited to detecting low frequency sounds. Comparison of the shapes of semicircular canals with lepidosaurs and archosauromorphs demonstrates that the semicircular canals of *Champsosaurus* are most similar to those of aquatic reptiles, suggesting that *Champsosaurus* was well adapted for sensing movement in an aquatic environment. This analysis also demonstrates that birds, non-avian archosauromorphs, and lepidosaurs possess significantly different canal morphologies, and represents the first morphometric analysis of semicircular canals across Diapsida.

## Introduction

Palaeoneurology, the study of the brain in the fossil record and how it has changed through time^1^, provides some of the best evidence for how extinct animals behaved and interacted with their environment. The behaviour and sensory abilities of extinct taxa are inferred based on the morphology of regions of the brain that are directly responsible for processing sensory information and forming behaviour. Other neural structures are often included in palaeoneurological studies, such as the cranial nerves and membranous labyrinth, which transmit sensory and motor information to and from the brain, and facilitate the sensation of movement and orientation, respectively. Estimations of sensory ability and behaviour based on the morphology of the brain are made possible by the principle of proper mass, which states that the size of a brain region dedicated to a specific function is directly correlated with the amount of processing power required to complete that function^2^. Therefore, regions of the brain that require more processing power tend to be larger to accommodate a greater number of neurons.

This correlation allows hypotheses to be made about the sensory ability of extinct animals based on the morphology of the brain;^3,4^ however, the brain endocast is not a perfect reflection of the brain in life, as it also represents other soft-tissue structures housed within the endocranial cavity that did not fossilize, such as the dura matter and vascular tissue^5^. Despite this, a description of the endocranial cavity of an extinct animal provides data that can be used to infer its neurosensory capabilities by comparison to closely related extant taxa, which allows for the formation of hypotheses regarding its behaviour and ecology^1^.

The morphology of the membranous labyrinth is also known to correlate with equilibrioception^6^ and auditory capabilities^7^, and is therefore a proxy for estimating these abilities^8^. Many recent studies have also found a correlation between the morphology of the semicircular canals and locomotor strategy and ecology, where phylogenetically distant lineages have convergent ear morphologies due to similar forms of locomotion and ecology^9–15^. Even though the correlation between canal morphology and ecology is well understood, it must be stated that the correlation is not perfect, and canal shape is known to be highly variable in some groups (e.g., sloths^10^ and saurischians^16^). Despite this, describing the morphology of the labyrinth in extinct taxa is likewise useful for inferring broad locomotor strategies or ecologies. Although *Champsosaurus* are widely accepted as aquatic^17–20^, a description of their endocranial anatomy and comparison of the inner ear with other taxa is necessary to determine if their sensory anatomy reflects the adaptations for aquatic habits seen in other aquatic reptiles.

Several studies have also demonstrated a strong phylogenetic signal in the morphology of semicircular canals in many different lineages^13^. This has important implications for *Champsosaurus* because the phylogenetic position of Choristodera within Neodiapsida is not well understood, where recent phylogenies have placed Choristodera in a polytomy with Archosauromorpha and Lepidosauromorpha^21,22^. Comparison of the inner ear of *Champsosaurus* with other neodiapsids could therefore provide novel information on the phylogenetic position of Choristodera.

Description of the brain, cranial nerves, and membranous labyrinth cannot be made directly in fossil taxa because these soft-tissue structures do not preserve; however, as they are usually encased within the osseous portion of the skull, which is frequently fossilized, aspects of their morphologies can be determined from their preserved passageways within the skull. The cavity that holds the brain is often referred to as the endocranial cavity, and is encased by the osseous chondrocranium and elements of the dermatocranium. Digital segmentation of this cavity can be used to produce a 3D structure (the brain endocast) that reflects the morphology of the endocranial cavity.

The membranous labyrinth of the inner ear, composed of sensory structures for both the auditory and equilibrium systems^23,24^, is also encapsulated by bone. In general, the bone sits quite close to the membranous labyrinth and the morphology of certain components, such as the semicircular canals, are therefore well represented in the morphology of the surrounding bone. 3D reconstructions of the cavity for the labyrinth (the endosseous labyrinth) are therefore used in palaeontological literature to hypothesize on the morphology of the membranous labyrinth in life, and to infer sensory abilities such as hearing and balance.

Historically, description of neurosensory structures relied on fragmentary material, or destructive sampling methods such as thin sectioning, but computed tomography (CT) scanning now allows for the 3D visualization of internal structures in intact specimens without causing damage to the specimen^1^.

The braincase of the Late Cretaceous reptile *Champsosaurus* is enigmatic due to the fragile nature of their skulls, hindering preservation. Fox^25^ provided a cursory description of the brain endocast, endosseous labyrinth, and cranial nerve passages of *Champsosaurus* based on fragmentary material. He was unable to provide an illustration of an intact endocast, or comment on the relative size of various regions of the brain, the shapes of the semicircular canals, or the paths of cranial nerve passages through the skull. There has been little discussion on the endocranial anatomy of *Champsosaurus* since Fox^25^, and the 3D morphology of these structures remains elusive, despite the valuable behavioural and ecological information that it could yield. The occurrence of *Champsosaurus* specimens in fluviolacustrine deposits suggests that these animals were highly aquatic, and its morphological similarity to the modern *Gavialis gangeticus* has led researchers to propose that *Champsosaurus* likely had a similar lifestyle^26^. Neurological evidence has yet to be considered.

Lu *et al*.^27^ described the endocranial anatomy of the Asian neochoristodere, *Ikechosaurus*, and commented on the possible presence of osteological correlates for turbinates in the nasal passage that may have facilitated thermoregulation. Choristodere thermoregulation was first proposed in *Champsosaurus* by Erickson^26^ simply due to the large surface area of the olfactory chambers of the nasal passages, but his description focused on fragmentary specimens and did not comment on the presence of turbinates. Turbinates in the neochoristodere nasal passage have not been reported since Lu *et al*.^27^, and it is not known whether this is a widespread feature of Neochoristodera. *Champsosaurus* is known to have occupied a wide latitudinal range, extending well into the polar region of the Canadian high arctic^28^ where contemporaneous crocodilians were absent^29^. This suggests that *Champsosaurus* was better able to tolerate the relatively cooler high-latitude temperatures than crocodilians, and *Champsosaurus* therefore may have had some form of thermoregulation.

**Figure 1:**
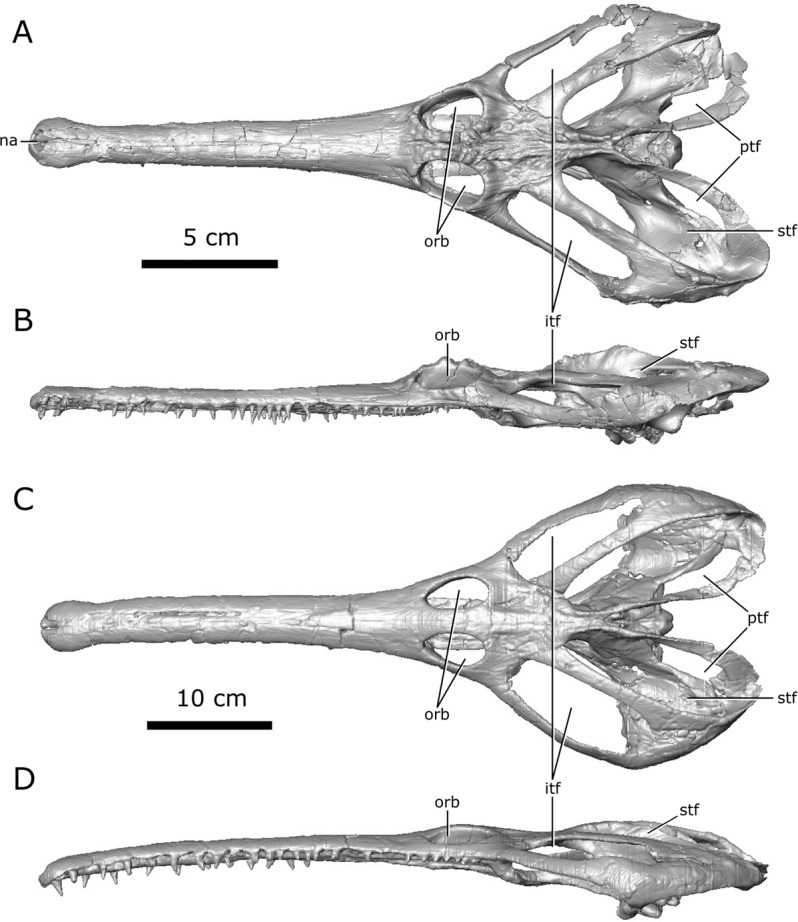
Digitized models of the skulls of *Champsosaurus lindoei* (CMN 8920) and *Champsosaurus natator* (CMN 8919) based on micro-computed tomography scanning. (**A)** CMN 8920 in dorsal view; (**B)** CMN 8920 in left lateral view; (**C)** CMN 8919 in dorsal view; (**D)** CMN 8919 in left lateral view. *Abbreviations: itf, infratemporal fenestra; na, narial opening; orb, orbit; ptf, post-temporal fenestra; stf, supratemporal fenestra*. Images generated in Amira 5.4.3 (https://www.fei.com/software/amira/) and processed in Inkscape 0.92 (https://inkscape.org/).

Here, the internal cranial anatomy of two *Champsosaurus* specimens, CMN 8920 (Canadian Museum of Nature; *C. lindoei*; Fig. 1) and CMN 8919 (*C. natator*), is described using CT scanning to provide data that can be used to make hypotheses about their sensory ability, behaviour, and ecology. A description of the endocranial anatomy using CT data, and comparisons with extant taxa, will provide novel data to either support or refute these previous hypotheses on the behaviour of *Champsosaurus*. Additionally, a description of the nasal passage of *Champsosaurus* will provide insight into the presence of turbinates, and allow for comment on the possible thermoregulatory ability of these animals.

## Materials and Methods

### Materials

CMN 8920 is a nearly complete skull, lacking jaws, with slight crushing of the right temporal arch. The specimen was found in 1953 in the mid-to-upper Campanian Dinosaur Park Formation, on the east branch of Little Sandhill Creek, Alberta, in what is now Dinosaur Provincial Park. It was first described by Russell^17^ as *C. natator*, but was later shown by Gao and Fox^30^ to belong to *C. lindoei* based on its relatively small size (approximately 24.3 cm in basal skull length), gracile snout, expanded narial bulla, and strait lower temporal bar.

CMN 8919 was found in 1917 in the Dinosaur Park Formation, near the same locality as CMN 8920, and approximately 10 m below the Bearpaw Formation (1917 field notes, CMN archives). This specimen consists of a well-preserved skull and mandible, the anterior half of the vertebral column, and both forelimbs. This specimen was formally described by Russell^17^, who attributed it to *C. natator*.

### Scanning and segmentation

CMN 8920 was scanned on October 19^th^, 2015 at the University of Texas CT facility (UTCT, Austin) with a voxel size of 60.5 μm at 200 kV and 0.3 mA. This produced 4579 jpeg files. Images were converted into tiff files and every other image was selected for segmentation. CMN 8919 was scanned on May 8^th^, 2018 at the Alta Vista Veterinary Hospital (Ottawa) using a 16-slice medical CT scanner with a voxel size of 0.5 mm at 135 kV and 75 mA. This produced 2542 jpeg images. Every other image was selected for segmentation. These data sets were loaded separately into Amira 5.4.3 (Visage Imaging GmbH, Berlin, Germany) for visualization and segmentation using the LabelFields module. Internal structures were segmented individually and rendered using the SurfaceView module, creating a colour coded model of the internal cavities for description and manipulation. The 3D models generated from this study will be made freely available online via MorphoSource (https://www.morphosource.org/Detail/SpecimenDetail/Show/specimen_id/25153) upon publication.

### Estimation of auditory perception

To estimate the mean best hearing frequency and best hearing frequency range of *Champsosaurus*, the length of the ventral portion of the membranous labyrinth (the pars inferior), which contains the sacculus, utriculus, and cochlea (or lagena^31^) of the endosseous labyrinth (see Supplementary Fig. S1), was compared to the extant dataset from Walsh *et al*.^7^. The dataset of Walsh *et al*.^7^ primarily used endocochlear duct length in their analysis, but for taxa that did not have a defined endocochlear duct (such as *Champsosaurus* in this study), Walsh *et al*.^7^ used the maximum length of the pars inferior (i.e., ventral to the lateral semicircular canal) in its place. This measurement for CMN 8920 was scaled to the length of the basicranium to account for skull size, and log transformed to normalize the data^7^. The length of the basicranium was measured from the posteriormost extent of the occipital condyle to the anteriormost edge of the basisphenoid, excluding the length of the cultriform process and the basipterygoid process (see Supplementary Fig. S1; P. Barrett, pers. comm., 2019). *Gerrhonotus multicarinatus* was excluded from this analysis because endocochlear duct length information was not available for that species. The dataset was subjected to ordinary least squares (OLS) linear regression in RStudio 1.1.456 (RStudio Inc.) and the scaled and transformed endocochlear length for CMN 8920 was inserted into the resulting equations to estimate hearing capability. OLS was chosen because other models, such as reduced major axis regression, become less accurate when the slope is far from ± 1, and OLS is known to be accurate when the measurement error associated with the dependent variable (hearing capability) is far greater than the variation in the independent variable (endocochlear duct length)^32^, as is the case here.

### Inferring ecology from the endosseous labyrinth

The following methods for inferring the ecology of *Champsosaurus* based on the morphology of the semicircular canals were modified from Dickson *et al*.^13^. The morphologies of the semicircular canals of CMN 8920 and CMN 8919 were compared to 59 species (61 specimens) of Lepidosauria and Archosauromorpha, and *Youngina* as an outgroup taxon (see Supplementary Tables S6-S8 for lists of species and specimen numbers). Turtles were included in this analysis due to the growing body of evidence suggesting that Pantestudines shares a close evolutionary history with early diapsids^33–36^.

Many studies have landmarked the centerline of the semicircular canals^9,13^; however, Mennecart and Costeur^37^ noted that landmarking the centerline does not take canal thickness into account, and that landmarks should instead be placed on the inner- and outer-most surfaces of the canals. This, unfortunately, cannot be done in this study because some taxa (including *Champsosaurus*) have some canals that lack a defined inner surface due to confluence with the pars inferior. Instead, the semicircular canals of the endosseous labyrinths were landmarked along the centerline using MorphoDig 1.2^38^. Left endosseous labyrinths were chosen because this side was best preserved in CMN 8920 and CMN 8919. When left endosseous labyrinths were not available for the comparative taxa, the right labyrinth was mirrored and used in place of the left. Curve handles and curve nodes were used to draw curves through the centerline of the three semicircular canals of each labyrinth. The first curve started at the center of the anterior ampulla, and ended at the junction of the anterior canal and the crus communis. The second curve started at the junction of the posterior canal with the crus communis, and ended at the center of the posterior ampulla. If the posterior ampulla could not be distinguished from the pars inferior, the landmarks ended where the posterior canal could no longer be distinguished from the pars inferior. The third curve started at the center of the lateral ampulla and ended where the lateral canal could no longer be differentiated from the pars inferior. Twenty evenly spaced landmarks were then projected onto each of these curves (total of 60 landmarks per labyrinth; see Supplementary Fig. S2) and exported in landmark file (*.lmk) format. Although the morphology of the crus communis is known to be ecologically significant^13^, this structure could not be landmarked in this analysis because some taxa (e.g., turtles) do not have a distinct osseous canal for the crus communis due to their enlarged pars inferior.

The landmark files from all 61 specimens were imported into RStudio 1.1.456 for analysis. The start and end landmarks of each curve were designated as type 1 landmarks^39^, and the remaining landmarks were designated as sliding semi-landmarks that were allowed to slide along the curve of the semicircular canal to minimize bending energy (see Supplementary Fig. S2). All landmarks and semi-landmarks were then rotated and scaled using General Procrustes Alignment in the R package *geomorph* 3.0.7^40^. The aligned landmarks were then projected into morphospace via a Principal Component Analysis (PCA) using the ‘plotTangentSpace’ function in *geomorph* to compare the morphology of the semicircular canals.

A phylogenetic tree was constructed based on the inferred relationships^41–47^ of these taxa to one another to evaluate phylogenetic signaling in the morphology of the semicircular canals. The occurrence data for these taxa were taken from the Paleobiology Database (paleobiodb.org) and the associated citations were verified for accuracy. Some extant species did not have known first occurrence dates (i.e., they do not have a known fossil record), so occurrence data of the genus were used in its place. Some extant genera did not have a known first occurrence date, so these taxa were entered with a first occurrence date of zero years before present. The tree was time-calibrated using the ‘cal3TimePaleoPhy’ tool in the R package *paleotree* 3.1.3^48^. Unsampled evolutionary history was estimated using the instantaneous per-capita sampling rate and instantaneous per-capita extinction rate derived from the ‘durationFreq’ function in *paleotree*. Instantaneous per-capita speciation rates were assumed to equal the instantaneous per-capita extinction rate^49^. A consensus tree was created using the ‘averageTree’ function in phytools based on 250 time-calibrated trees produced by cal3TimePaleoPhy. The consensus time-calibrated phylogeny was projected onto the PCA using the ‘plotGMPhyloMorphoSpace’ function in *geomorph* to visualize the change in canal shape across evolutionary time.

Bloomberg’s multivariate K statistic^50^ was calculated for centroid size and landmark coordinates using the consensus time-calibrated phylogeny and the *geomorph* function ‘physignal’ with 1000 permutations to evaluate phylogenetic signal on centroid size and canal morphology. Bloomberg *et al*.^51^ surveyed published datasets and determined that phylogenetic signal could be detected (*p* < 0.05) using the K statistic in 92% of studies that involved 20 or more taxa. This suggests that the number of taxa used here (59) is adequate to assess phylogenetic signal.

The included taxa were divided into five major ecological groups based on the medium that they interact with most during locomotion: aerial taxa that fly; aquatic taxa that spend the majority of their time in water; arboreal taxa that live predominantly in trees; fossorial taxa that dig through soil; and terrestrial taxa that predominantly live on land. For fossil taxa (e.g., non-avian dinosaurs) the ecological groups were assigned based on inferred ecology in the published literature. Analysis of Covariance (ANCOVA) and phylogenetic generalized least squares (PGLS) were performed using the ‘procD.lm’ and ‘procD.pgls’ functions in *geomorph*, respectively, to determine whether taxa separate in the morphospace based on ecology. These two tests were used in tandem because, together, they illustrate the relationship between ecology and canal morphology when phylogenetic lineages are considered as either independent (ANCOVA) or dependent (PGLS)^13^.

A canonical variates analysis (CVA) was then run on the first 17 PC scores of all taxa except *Champsosaurus*, and the *Champsosaurus* specimens were then projected onto the CVA to predict their ecology. Only the first 17 PC scores were included in the CVA because this is the minimum number of PC scores needed to describe 95% of the total variation in canal morphology, and because removing the smaller PC scores eliminates subtle variances in canal morphology that may be due to measurement error^13^. The CVA was run using the ‘CVA’ function in the R package *Morpho* 2.6^52^. Ninety-five percent confidence ellipses were generated around the ecological groups within the CVA morphospace. *Typhlops hypomethes* was removed from the dataset prior to the CVA because it was the only fossorial taxon included in the analysis, and the function ‘CVA’ cannot accept a group of n = 1. The posterior probability of group membership was calculated for both *Champsosaurus* specimens by calculating the Mahalanobis distances of the specimens from each group mean, and comparing those distances to within-group distances, with 10 000 resampling permutations. Specimens that plotted farther away from a group mean than 95% of within-group distances were considered significantly different from that ecological group. Log-likelihood estimations were calculated to determine ecological group assignment for *Champsosaurus*. All tests that calculated a p-value were subjected to Bonferroni correction to account for multiple comparisons.

## Results

### *Champsosaurus* brain endocast

CMN 8920 (*Champsosaurus lindoei*) was scanned at a higher resolution than CMN 8919 (*C. natator*), and therefore more accurately illustrates details of the endocranial anatomy of *Champsosaurus*. As such, CMN 8920 forms the basis of this description, and only notable differences with CMN 8919 will be discussed. Taphonomic deformation of both braincases is minimal, so we infer that the reconstructed endocasts accurately reflect the living brain endocast morphology.

**Figure 2:**
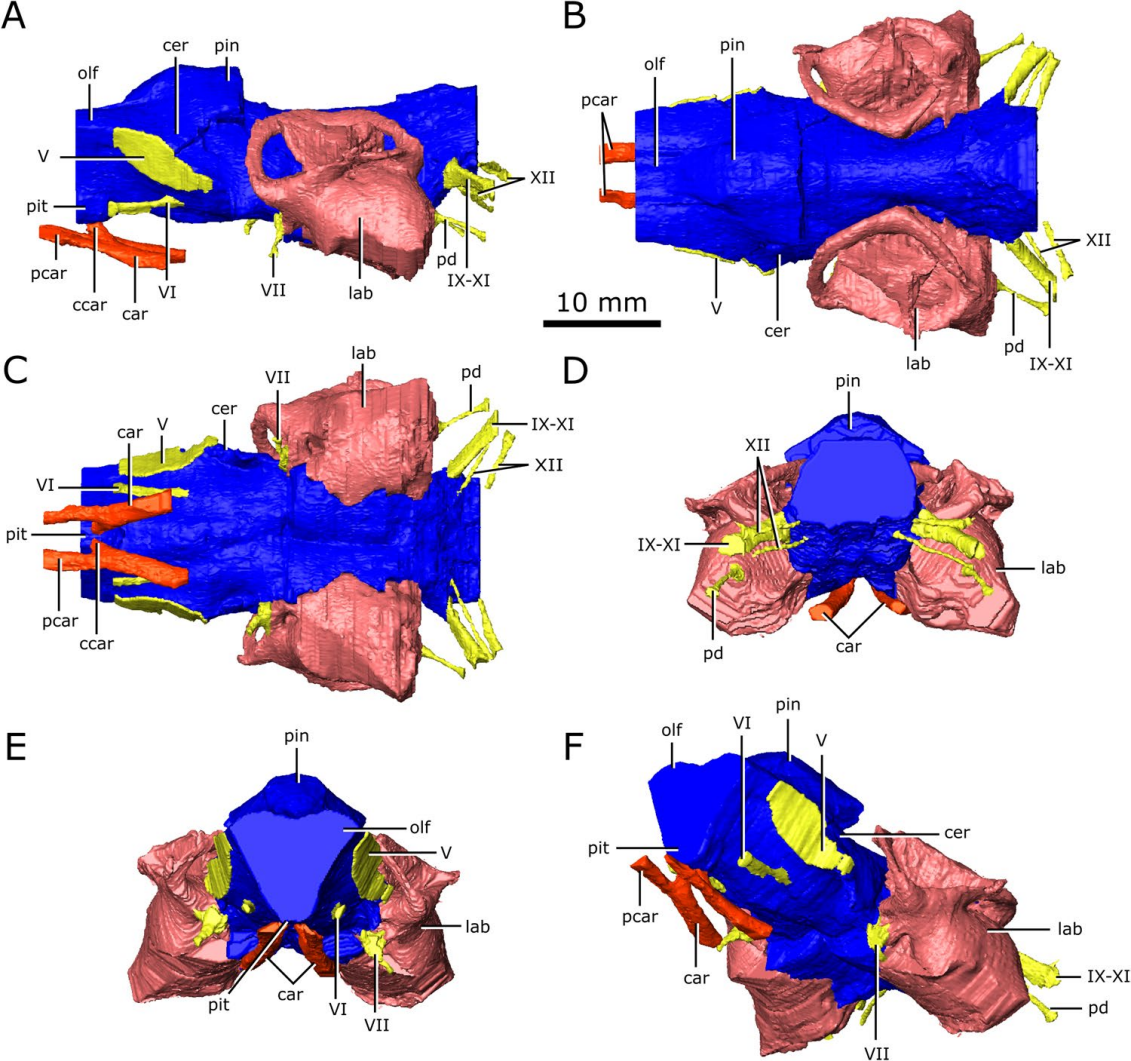
Reconstruction of the endocranial anatomy of *Champsosaurus lindoei* (CMN 8920). (**A)** left lateral view; (**B)** dorsal view; (**C)** ventral view; (**D)** posterior view; (**E)** anterior view; (**F)** left anterolateroventral view. The brain endocast is illustrated in blue, endosseous labyrinth in pink, cranial nerves in yellow, and carotid artery in red. *Abbreviations: car, carotid arteries; ccar, cerebral branch of the carotid arteries; cer, cerebrum; lab, endosseous labyrinth; olf, base of the olfactory lobes; pcar, palatine branch of the carotid arteries; pd, canal for the parilymphatic duct; pin, pineal body; pit, pituitary fossa; IX-XI, canal for cranial nerves IX, X, and XI; XII, canal for cranial nerve XII; V, opening for cranial nerve V; VI, canal for cranial nerve VI; VII, canal for cranial nerve VII*. Images generated in Amira 5.4.3 (https://www.fei.com/software/amira/) and processed in Inkscape 0.92 (https://inkscape.org/).

**Figure 3:**
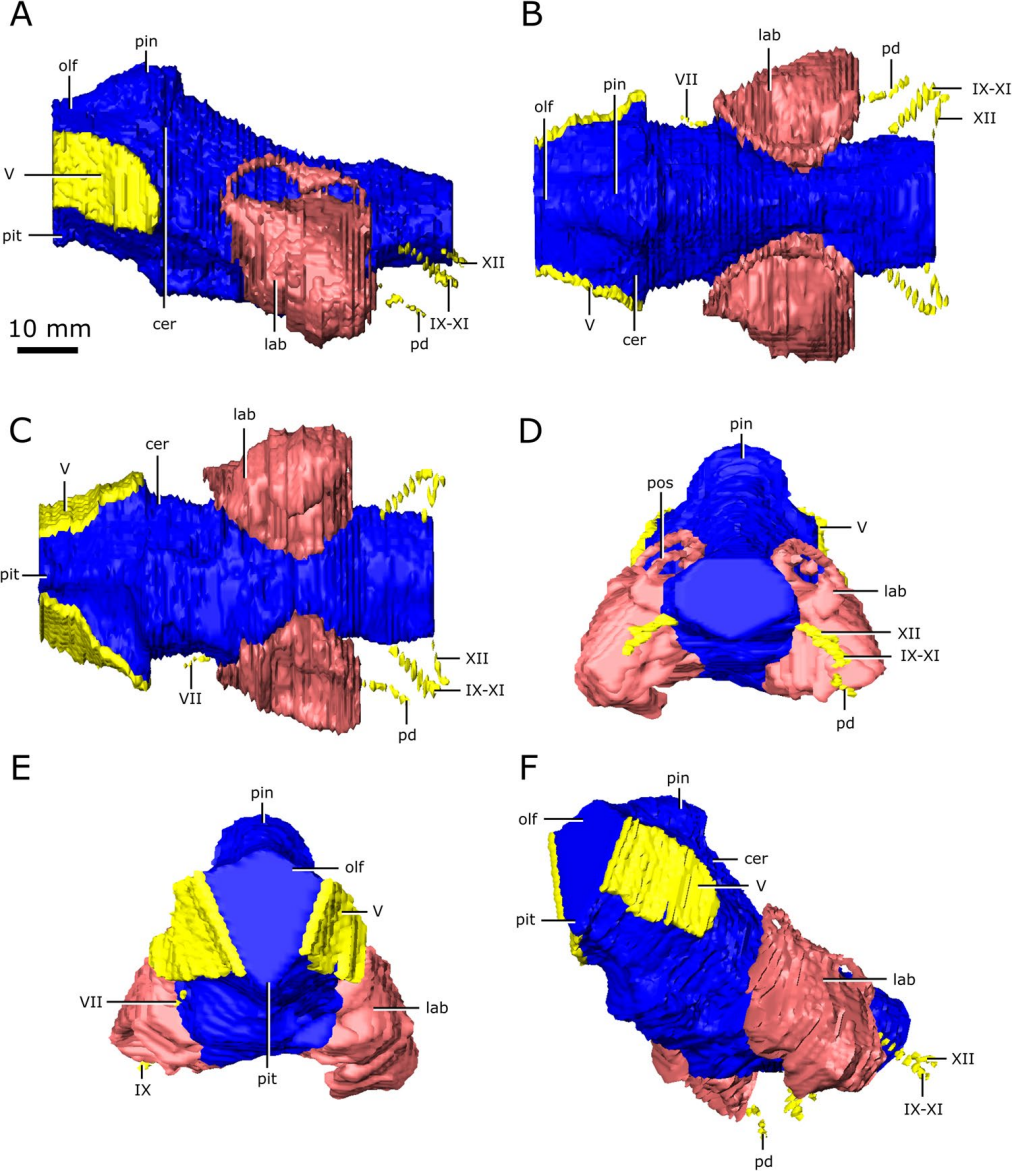
Reconstruction of the endocranial anatomy of *Champsosaurus natator* (CMN 8919). (**A)** left lateral view; (**B)** dorsal view; (**C)** ventral view; (**D)** posterior view; (**E)** anterior view; (**F)** left anterolateroventral view. The brain endocast is illustrated in blue, endosseous labyrinth in pink, cranial nerves in yellow, and carotid artery in red. *Abbreviations: cer, cerebrum; lab, endosseous labyrinth; olf, base of the olfactory lobes; pd, canal for the parilymphatic duct; pin, pineal body; pit, pituitary fossa; IX-XI, canal for cranial nerves IX, X, and XI; XII, canal for cranial nerve XII; V, opening for cranial nerve V; VII, canal for cranial nerve VII*. Images generated in Amira 5.4.3 (https://www.fei.com/software/amira/) and processed in Inkscape 0.92 (https://inkscape.org/).

The brain endocast of CMN 8920 is narrow both mediolaterally and dorsoventrally, and does not show flexure (Fig. 2). In contrast, the brain endocast of CMN 8919 shows distinct cerebral and pontine flexures, an observation that is corroborated by fragmentary specimens of *C. natator* (ROM 688). The walls around the midbrain and hindbrain are well-ossified and provide good anatomical detail, but the lateral and ventral walls around the olfactory stalks did not ossify, and the exact shape of the olfactory stalks therefore cannot be determined. The ossified braincase of CMN 8920 is approximatley 32 mm long, but impressions of the olfactory stalks of the brain on the ventral surface of the parietals and frontals show that the entire brain cavity is 67 mm long (Fig. 3). The olfactory stalks are substantial (approximately 37 mm long from the anterior margin of the pineal body) and occupied approximately 55% of the total length of the brain endocast.

**Figure 4:**
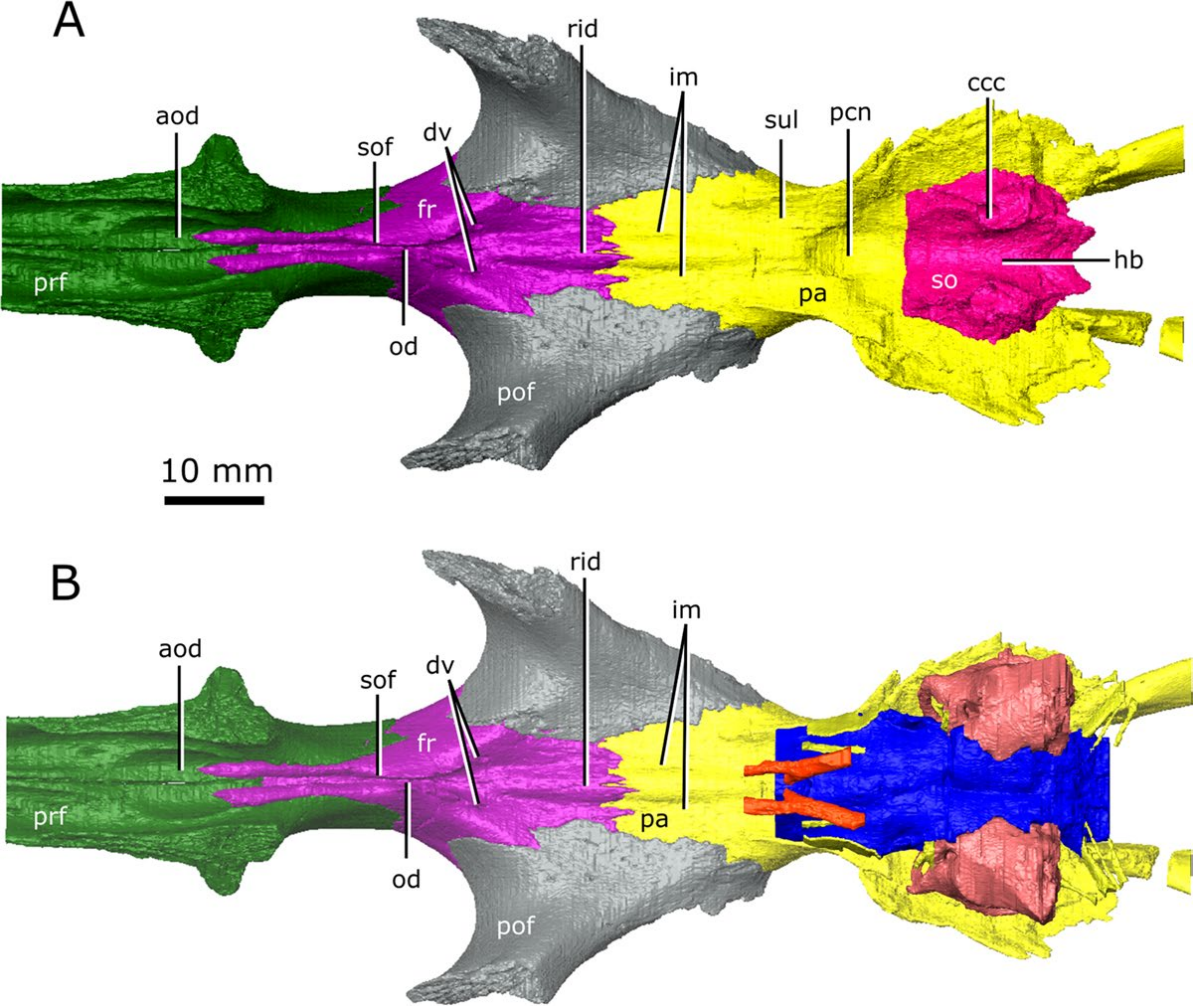
The isolated braincase roof (prefrontals, frontals, postfrontals, parietals, supraoccipital) of CMN 8920. (**A**) Ventral view of the isolated braincase roof; (**B)** Ventral view of the braincase roof with the segmented brain endocast (blue), endosseous labyrinth (pink), cranial nerves (yellow), and carotid arteries (red) in position. The braincase roof is slightly faded. *Abbreviations: aod, anterior of olfactory duct; ccc, canal for the crus communis; dv, diploic vein foramen; fr, frontal; hb, roof of the hindbrain; im, impressions of the olfactory tracks; od, olfactory duct; pcn, parietal concavity for the pineal body; pa, parietal; pof, postfrontal; prf, prefrontal; rid, ridge seperating the paired olfactory tracts; so, supraoccipital; sof, subolfactory flange; sul, area inundated with sulci*. Images generated in Amira 5.4.3 (https://www.fei.com/software/amira/) and processed in Inkscape 0.92 (https://inkscape.org/).

The ossified braincase of CMN 8919 is approximately 66 mm long and the entire brain cavity is approximately 126 mm long (Fig. 3). The olfactory stalks of the brain of CMN 8919 are also elongate, measuring approximatley 65 mm long from the anterior margin of the pineal body, and occupy approximatley 51% of the length of the brain endocast. Two foramina lead into the ventral surface of the frontals in the impression left by the anterior-most extent of the olfactory stalks of CMN 8920. CT data reveal that these foramina fork and dissipate into the cortical bone of the frontals, suggesting that they are vascular and carried diploic veins (Fig. 4; dv)^4^.

The brain endocasts of both specimens are fully enclosed by bone posterior to the olfactory stalks, preserving the morphology of the midbrain and hindbrain. The lateral, posterior, and ventral walls of the pituitary fossa are formed by the basisphenoid, but the anterior wall did not ossify. The pituitary fossa of both specimens is shallow, wide, and lacking details such as sulci, suggesting the pituitary gland would not have occupied the entirety of this space, and was supported by a thick layer of dura matter.

Posterior to the pituitary fossa, the endocast expands dorsally into a large concavity in the ventral surface of the parietals (Fig. 2; Fig. 3; pin). Russell^17^ interpreted this expanson as a portion of the cerebellum, but based on its position in the midbrain, it appears to be the pineal expansion that is also present in other neodiapsids (e.g., phytosaurs^53^). The CT data of CMN 8920 show that the dorsal surface of the brain endocast anterior to the pineal expansion possesses sulci, suggesting that the brain pressed close to the skull in this region (Fig. 4; sul). The CT data of CMN 8919 are of too low resolution to capture these fine details; however, these sulci are also present on fragmentary specimens of *C. natator* (CMN 8921; CMN 8922; CMN 32579; ROM 688), suggesting that sulci in this region are widely present in *Champsosaurus*.

**Figure 5:**
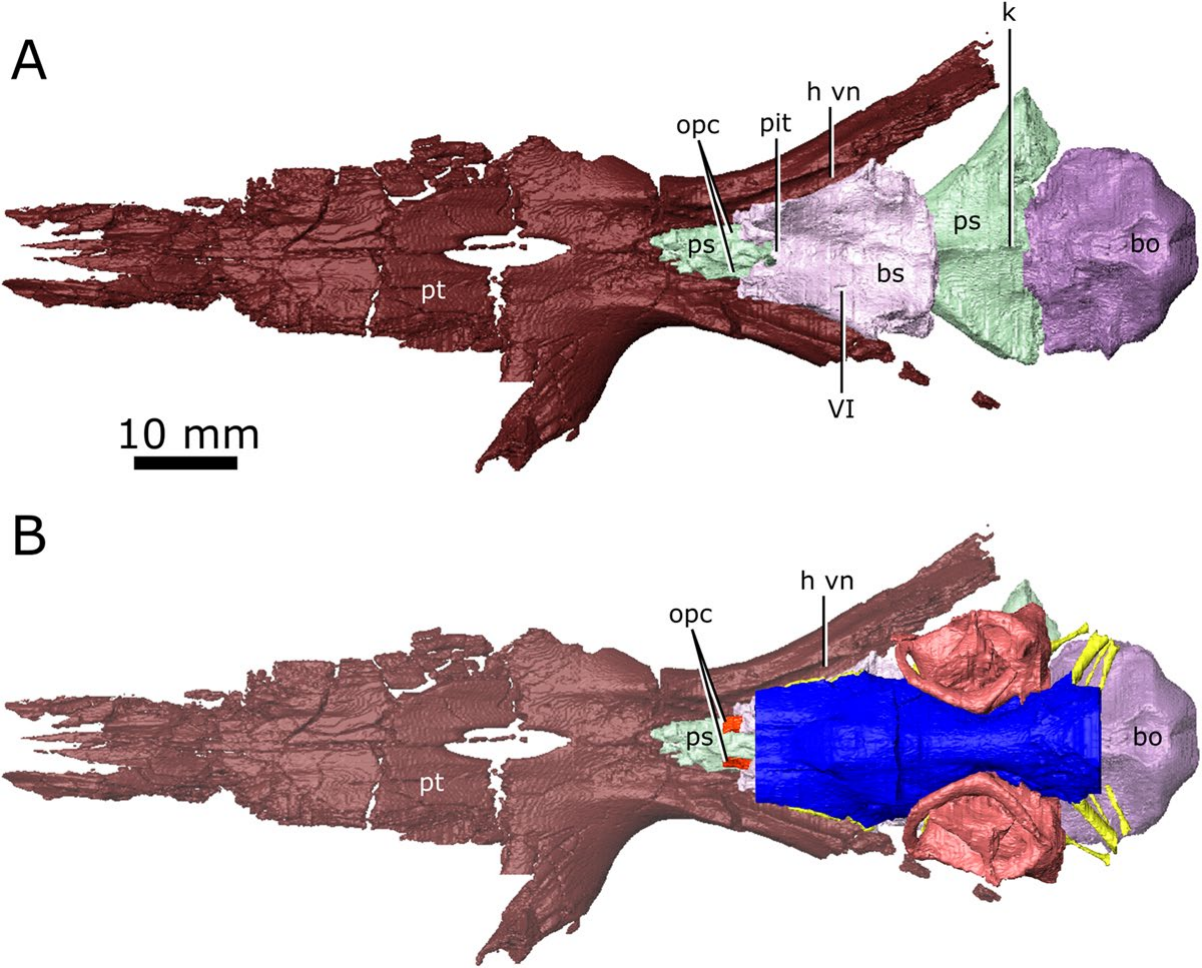
The isolated braincase floor (pterygoids, basisphenoid, parasphenoid, and basioccipital) of CMN 8920. (**A**) Dorsal view of the isolated braincase floor; (**B**) Dorsal view of the braincase floor with the segmented brain endocast (blue), endosseous labyrinth (pink), cranial nerves (yellow), and carotid arteries (red) in position. The braincase floor is slightly faded. *Abbreviations: bo, basioccipital; bs, basisphenoid; hvn, trough for the lateral head vein; k, parasphenoid keel; opc, opening for the palatine branch of the carotid artieries; pit, pituitary fossa; ps, parasphenoid; pt, pterygoid; VI, exit for cranial nerve VI*. Images generated in Amira 5.4.3 (https://www.fei.com/software/amira/) and processed in Inkscape 0.92 (https://inkscape.org/).

Posterior to the pineal expansion, the brain endocast narrows both dorsoventrally and mediolaterally, and is flanked on either side by the endosseous labyrinths (Fig. 2; Fig. 3; lab). The optic lobes, cerebellum, and flocculus are not evident in the brain endocast of either CMN 8920 or CMN 8919, and were likely small and covered by thick dura matter. The parasphenoid forms the floor of the braincase medial to the endosseous labyrinths (Fig. 5). A strong sagital keel is present on the dorsal surface of the parasphenoid and posterior-most portion of the basisphenoid that axially bisects the ventral portion of the endocranial cavity that housed the brain stem (Fig. 5; k). The keel is evident in the CT data for CMN 8920 and is also present in fragmentary specimens (CMN 8922; ROM 688), but it is not seen in the CT data for CMN 8919, likely due to a combination of low scan resolution and damage during preperation. Fox^25^ described this region of the endocast as a deep basin, but did not comment on the presence of a dorsal keel on the parasphenoid. Posterior to the parasphenoid, the brain endocast is floored by the basioccipital and expands dorsally and mediolaterally, but does not reach the same width or height as it does anterior to the auditory system. The brain endocast extends posteriorly and opens to the foramen magnum.

### Cranial nerves

**Figure 6:**
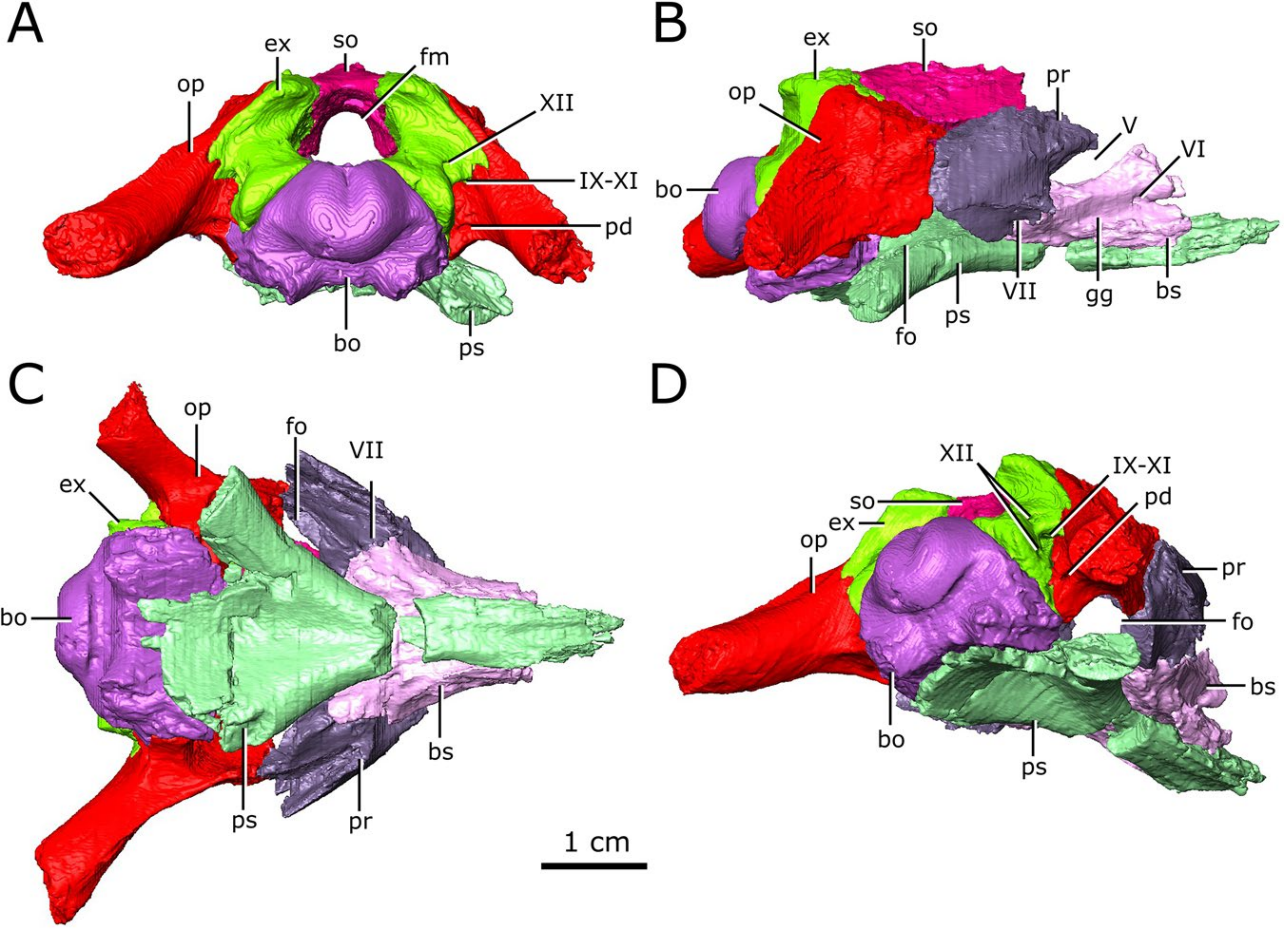
Isolated chondrocranial elements and parasphenoid of CMN 8920. (**A**) posterior view; (**B**) right lateral view; (**C)** ventral view; (**D**) posterolateroventral view. *Abbreviations: bo, basioccipital; bs basisphenoid; ex, exoccipital; fm, foramen magnum; fo, fenestra ovalis; gg, depression for the gasserian ganglion; op, opisthotic; pd, foramen for the perilymphatic duct; pr, prootic; ps, parasphenoid; so, supraoccipital; Roman numerals indicate the foramina of the corresponding cranial nerves*. Images generated in Amira 5.4.3 (https://www.fei.com/software/amira/) and processed in Inkscape 0.92 (https://inkscape.org/).

The cranial nerve passages of CMN 8919 could not be observed due to low scanning resolution, but were clearly visible in CMN 8920. The following description is based predominantly on the cranial nerve passages of CMN 8920 (Fig. 6).

The olfactory duct is preserved between the subolfactory flanges (= crista cranii of some other diapsid groups, such as lepidosaurs^54^) on the ventral surface of the frontals, extending from the posteriormost portion of the olfactory chambers of the nasal passages to the region occupied by the olfactory bulbs of the brain (Fig. 4; od). As mentioned previously, the anterior braincase did not ossify in *Champsosaurus*, and the pathways for cranial nerves II-IV are not preserved. Dorsal to the pituitary fossa, there is a large, paired opening in the walls of the braincase that is bordered by the parietal dorsally, and the basisphenoid ventrally (Fig. 6; V). This opening likely carried the trigeminal nerve (CN V) as it exited the endocast, but does not show evidence for the divergence of CN V into its three rami, CN V_1_ (ophthalmic), CN V_2_ (maxillary), and CN V_3_ (mandibular), suggesting that this divergence would have occurred outside of the boney braincase. Supporting the extra-cavity split of CN V is a shallow depression in the lateral wall of the basisphenoid ventral to the opening for CN V that may have held the gasserian ganglion (Fig. 6; gg). A shallow groove extends posteriorly from the opening for CN V along the lateral surface of the parietal and neomorph towards the pterygoquadrate foramen that may represent the path of CN V_3_, although it is also possible that the groove represents the path of the stapedial artery (see Vasculature section below for discussion). The only preserved portion of the pathway for CN V_2_ is in the snout, originating in the ventral rim of the orbit (see Supplementary Figure S3) and extending anteriorly through the maxilla and premaxilla to the tip of the snout. The canal branches repeatedly along its length, where the branches lead to the outer surface of the skull and likely carried sensory nerves to innervate the snout. CN V_1_ would have extended anterodorsally to inervate the orbit and integument of the snout^55^, but was supported by soft tissue and its pathway was not preserved.

Posterior to the pituitary fossa, a paired canal, which likely carried the abducens nerve (CN VI), extends anteriorly from the floor of the brain cavity and exits through the lateral surface of the basisphenoid (Fig. 6; VI). A paired canal, which likely carried the facial nerve (CN VII), extends ventrolaterally from the floor of the endocast anterior to the labyrinth (Fig. 6; VII). The left CN VII canal passes between the basisphenoid and the prootic for its entire length, but the right nerve canal exits directly through the prootic, only contacting the basisphenoid for a small portion anteriorly near the ventral surface of the skull. There is no osseous canal for the vestibulocochlear nerve (CN VIII). Instead, the otic capsule communes to the brain cavity through a broad opening, suggesting that the canal for CN VIII and the majority of the medial wall of the otic capsule were cartilagenous in life. The absence of an osseous canal for CN VIII in CMN 8920, CMN 8919, and other specimens of *Champsosaurus*^25^ suggests that a cartilagenous medial wall to the otic capsule was widely present in *Champsosaurus*.

A canal originates at the posterior wall of the otic capsule and extends posteriorly through the opisthotic. We previously interpreted this as the passage for the glossopharyngeal nerve^25,56^ (CN IX). More likely, this canal carried the perilymphatic duct (Fig. 2; Fig. 3; pd) extending posteriorly from the otic capsule^57^, and CN IX would instead have exited with the vagus (CN X) and accessory (CN XI) nerves, as seen in some modern reptiles such as crocodilians^1^. A canal extends ventrolaterally from the endocast and exits between the opisthotics and exoccipitals (Fig. 2; Fig. 3; Fig. 6; IX-XI). The relatively large diameter, and the position of the canal between the opithotic and exoccipital, suggests that it is the vagal foramen and would have carried CN IX, CN X, and CN XI^57–59^. Posterior to the canal for CN IX, CN X, and XI, two paired, narrow canals that carried branches of hypoglossal nerve (CN XII) exit ventromedially at the opening for the foramen magnum and extend posterolaterally through the exoccipitals (Fig. 2; Fig. 3; Fig. 6; XII).

### Endosseous labyrinth

**Figure 7:**
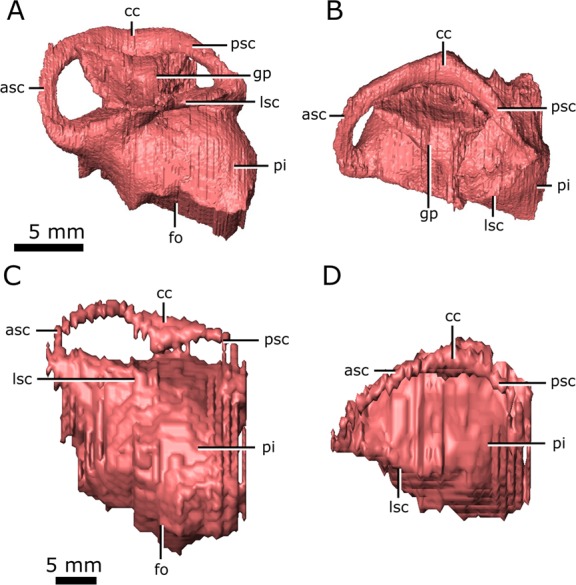
Left endosseous labyrinth of *Champsosaurus lindoei* (CMN 8920) in: (**A**) lateral view; and (**B**) dorsal view, and *Champsosaurus natator* (CMN 8919) in: (**C**) lateral view; and (**D**) dorsal view. *Abbreviations: asc, anterior semicircular canal; cc, crus communis, fo, fenestra ovalis; gp, unossified gap between the prootic, opisthotic, and supraoccipital; lsc, lateral semicircular canal; pi, pars inferior; psc, posterior semicircular canal*. Images generated in Amira 5.4.3 (https://www.fei.com/software/amira/) and processed in Inkscape 0.92 (https://inkscape.org/).

Dorsally, the anterior and posterior semicircular canals appear as distinct, tubular structures (Fig. 7; asc; psc); however, the entire lateral canal of CMN 8919 is confluent with the dorsolateral surface of the pars inferior, as is the anterior half of the lateral canal of CMN 8920 (Fig. 7; lsc). This suggests that the medial wall of the lateral semicircular canal was poorly ossified in *Champsosaurus*, and that regions of the labyrinth, such as the lateral canal, would have been supported by soft tissue and cartilage within the otic capsule in life. Additionally, the prootic, opisthotic, and supraoccipital fail to contact each other lateral to the labyrinth in CMN 8920, creating a cavity that projects dorsolaterally from the pars inferior (Fig. 7; gp). This cavity is absent in CMN 8919, suggesting that the otic region was better ossified in larger animals, although the lateral canal is no better preserved in CMN 8919 than it is in CMN 8920. The angle of the lateral canal from the long axis of the skull varies considerably between CMN 8920 and CMN 8919, where the lateral canal is oriented approximately −15.8° from the long axis of the skull in CMN 8920 (tilted anteroventrally), and approximately 13.3° from the long axis of the skull in CMN 8919 (tilted anterodorsally).

The anterior ampullae are small, but can be distinguished as an enlargement of the canals at their anteriormost extent. The posterior ampulla is not evident in the endosseous labyrinth, but would have been located at the posteriormost extent of the posterior canal. In CMN 8920 and CMN 8919 the pars inferior forms a bulbous cavity ventral to the semicircular canals. The fenestra ovalis is located on the ventral surface of the pars inferior (Fig. 7; fo), with no portion of the endosseous labyrinth extending ventral to it (e.g., the cochlear duct), possibly due to the dorsoventrally flattened skull profile of *Champsosaurus*. This is in contrast to the morphology of most reptiles, where the fenestra ovalis is located on the lateral surface of the pars inferior and the cochlear duct extends ventral to it. There is no clear separation between the cochlear duct and the sacculus in CMN 8920 and CMN 8919.

### Vasculature

The passages for the internal carotids are not visible in the CT data for CMN 8919, and the morphology of these arteries is based entirely on CMN 8920. The internal carotids entered the skull through passages on the ventral surface of the skull that passed between the contact of the parasphenoid and pterygoid, and extended anterodorsally towards the pituitary fossa (Fig. 2; car). Ventral to the pituitary fossa, the canals fork, where the dorsal branch carried the cerebral artery that opened into the pituitary fossa (Fig. 2; ccar), and the ventral branch carried the palatine artery, continuing anteriorly until it opens on the dorsal surface of the pterygoid, anterior to the basisphenoid (Fig. 2; pcar). The path of the palatine artery canals anterior to the basisphenoid cannot be determined due to incomplete ossification of this region.

The lateral head vein sits in a deep trough formed by the quadrate ramus of the pterygoid, and the lateral wall of the basisphenoid (Fig. 5; h vn). Fox^25^ stated that a channel is imprinted on the lateral wall of the clinoid process of the basisphenoid that drained the orbital sinus into the lateral head vein, but this channel is not present in CMN 8920 and CMN 8919. Additionally, Fox^25^ described a foramen penetrating the quadrate ramus of the pterygoid that carried the lateral head vein, but no such foramen is seen in either CMN 8920 or CMN 8919. Instead, the lateral head vein appears to have extended posteriorly along the trough formed by the pterygoid and basisphenoid, and exited the skull lateral to the fenestrae ovales. Fox^25^ also suggested that a groove imprinted into the lateral surface of the parietal and neomorph leading from the exit for CN V to the pterygoquadrate foramen is an impression of the stapedial artery. If Fox’s^25^ interpretation is correct, the stapedial artery would have divided into the superior and inferior branches anterior to the opening for CN V^25^.

### Nasal cavity

**Figure 8:**
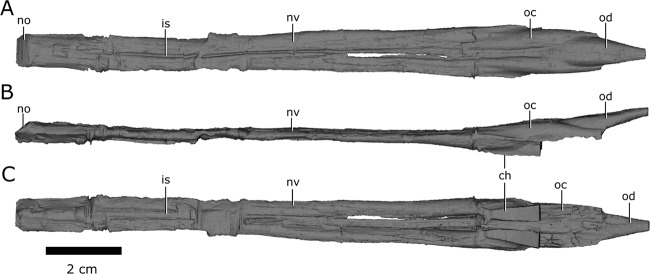
Left and right nasal passages of *Champsosaurus lindoei* (CMN 8920), in (**A**) dorsal view; (**B**) left lateral view; and (**C**) ventral view. *Abbreviations: ch, choana; is, ridge indicating the internarial septum; no, narial opening; nv, nasal vestibule; oc, olfactory chamber; od, olfactory duct*. Images generated in Amira 5.4.3 (https://www.fei.com/software/amira/) and processed in Inkscape 0.92 (https://inkscape.org/).

Like the snout of CMN 8920, the nasal passage is highly elongate, measuring approximately 14 cm from the anteriormost extent of the narial opening to the posteriormost extent of the olfactory chambers (Fig. 8). An ossified internarial septum is absent, but longitudinal ridges at the confluence of the left and right vomers, and the midline of the internarial and nasal, suggest it would have been present as cartilage in life.

The nasal passages are ovoid in cross-section, each measuring approximately 0.8 cm in width, and 0.45 cm in height. The floor of the nasal passage is severely damaged, and several fragments of bone have been displaced dorsally into the nasal passage. The fragmentation is most prominent midway along the nasal passage, and along the posteriormost extent of the olfactory chambers. The dorsolateral walls of the olfactory chambers are well-preserved, revealing that the olfactory chambers are also elongate, measuring approximately 2.9 cm in length (Fig. 8; oc). The choanae open ventrally from the anterior floor of the olfactory chambers between the palatine laterally and the vomer medially (Fig. 8; ch).

The single olfactory duct extends posterodorsally from the olfactory chambers towards the brain endocast, between the paired subolfactory flanges of the frontals (Fig. 4; Fig. 8; od), and narrows posteriorly to form the passage for the olfactory nerve (CN I). With the exception of fragmentation of the floor of the nasal passage, the osseous walls of the nasal passage are smooth, and there are no ridges present that are suggestive of turbinates.

### Auditory capabilities

**Figure 9:**
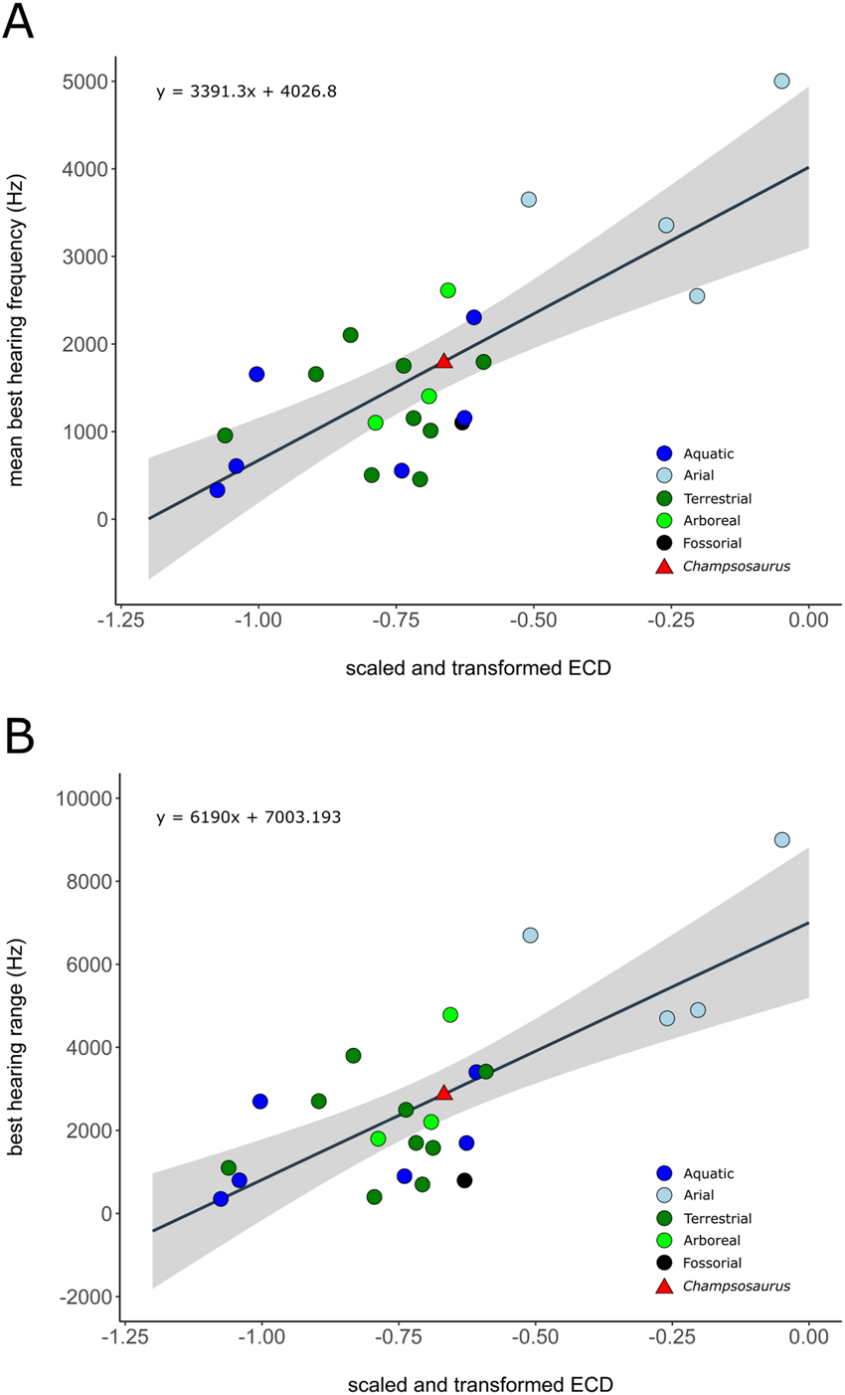
Correlation between scaled and transformed endocochlear duct length (ECD) and (**A**) mean best hearing frequency (y = 3391.3x + 4026.8; r^2^ = 0.5825; *p* = 2.28e-05); (**B**) best hearing range (y = 6190x + 7003.193; r^2^ = 0.5521; *p* = 4.875e-05) with ecologies coloured separately. Grey area indicates the 95% confidence interval of the regression line. Extant data from Walsh *et al*. (2009). Red triangle indicates the predicted value for *Champsosaurus lindoei* (CMN 8920).

The mean best hearing frequency and best hearing range were plotted against the endocochlear duct length for the extant taxa from the data provided by Walsh *et al*.^7^. The length of the scaled and transformed pars inferior for CMN 8920 (−0.65698) was inserted into the derived equations of the regression lines (Fig. 9), resulting in a best hearing frequency of 1798.8 Hz, and a best hearing range of 2936.5 Hz (overall best hearing range: 330.6–3267.1 Hz).

### Geometric morphometrics of the semicircular canals

**Figure 10:**
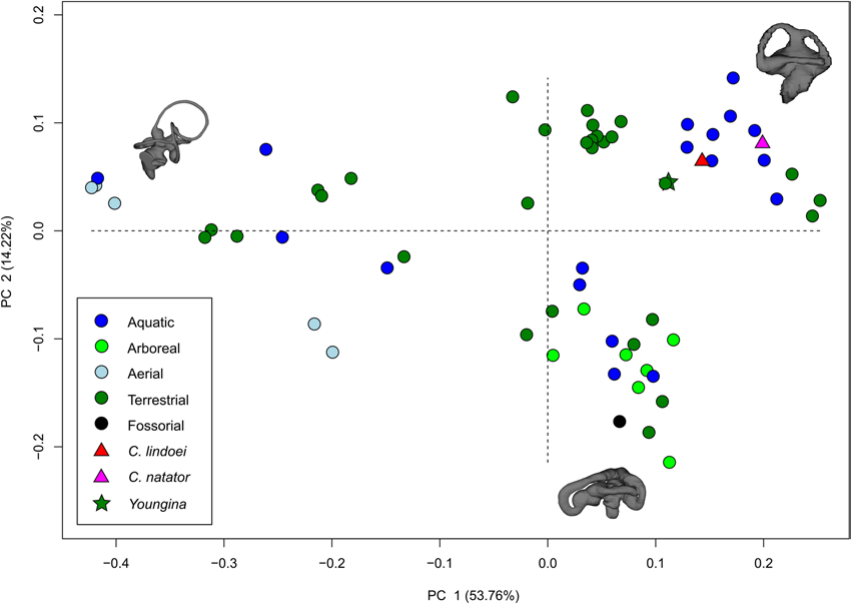
PC 1 vs PC 2, representing 67.98% of the total variation. Taxa are colour coded based on ecology. End-point morphologies: top right, *Tomistoma schlegelii*; bottom, *Aheatulla nasuta*; top left, *Passer domesticus*.

**Figure 11:**
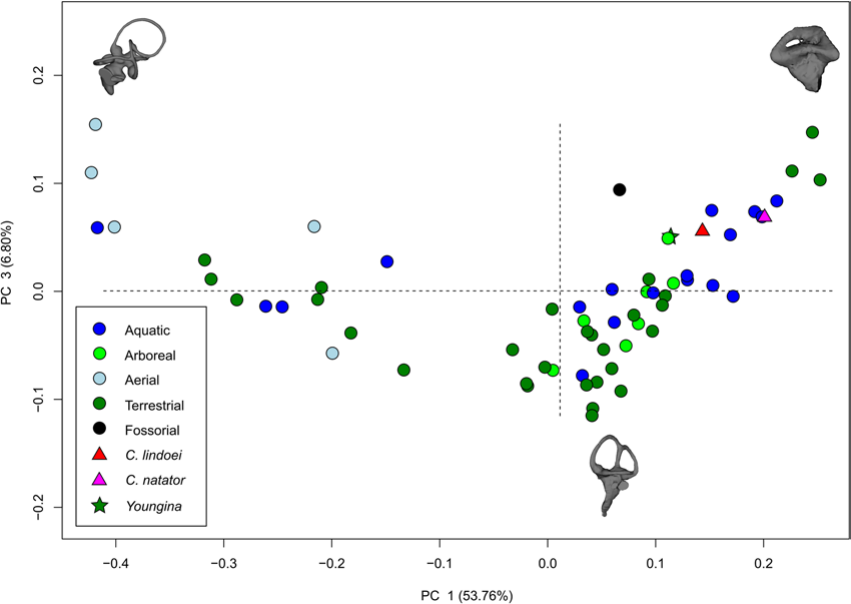
PC 1 vs PC 3 representing 60.56% of the total variation. Taxa are colour coded based on ecology. End-point morphologies: top left, *Passer domesticus*; bottom, *Erlikosaurus andrewsi*; top right, *Manouria emys*.

The PCA produced 62 PC axes, but only axes 1 through 3 will be discussed here (cumulative variation = 74.78%) because the remaining PC axes account for relatively little variation (<5% each). Plotting PC1 against PC2 (Fig. 10) shows that PC1 (53.76% of variation) mostly represents curvature of the anterior semicircular canal, angling of the posterior canal relative to the rest of the labyrinth, curvature of the lateral canal, and angling of the lateral canal relative to the rest of the labyrinth. Positive PC1 values represent a dorsoventrally compressed, less curved anterior canal, a posterodorsally angled posterior canal, and an anterodosally angled lateral canal that is less curved anteriorly. Negative PC2 values represent a dorsoventrally elongated and curved anterior semicircular canal, an anterodorsally angled posterior canal, and an anteroventrally angled lateral canal that is more curved anteriorly. Birds, which plot towards PC1 negative, have the most extreme condition, with an anterior semicircular canal that arcs over the posterior semicircular canal and enters the crus comunis posteriorly, a posterior canal that is strongly angled anteroventrally relative to the rest of the labyrinth, and an anteroventrally angled lateral canal relative to the rest of the labyrinth. PC2 (14.22% of variation) mostly represents anteroposterior elongation and out-of-plane curvature (torsion) of the anterior semicircular canal, curvature of the posterior canal, and torsion of the lateral canal. Positive PC2 values represent anteroposterior compression and medial torsion of the anterior canal, a more tightly curved posterior canal, and less torsion in the lateral canal. Negative PC2 values represent anteroposterior elongation and lateral torsion of the anterior canal, a more widely curved posterior canal, and greater torsion of the lateral canal. PC3 (6.80% of variation; Fig. 11) represents a combination of curvature of the anterior canal, angle of the anterior and posterior canals relative to one another, and the curvature of the lateral canal. Positive PC3 values indicate a smaller, more eliptical anterior canal, a more acute angle between the anterior and posterior canals, and a more widely curved lateral canal. Negative PC3 values indicate a larger, more curved anterior canal, a more abtuse angle between the anterior and posterior canals, and tighter curvature of the lateral canal.

When visualizing PC1 vs PC2 (Fig. 10), three distinct groups are formed that appear separated by phylogeny: Aves (left), Lepidosauria (bottom right), and non-avian archosauromorphs (top right). Projecting a phylogeny (see Supplementary Fig. S4) onto PC1 vs PC2 (see Supplementary Fig. S5) clearly illustrates these groups, suggesting that phylogeny strongly influences grouping in this PCA.

Phylogenetic signal was less than expected under Brownian motion (K < 1; see Supplementary Table S1) in both centroid size and canal morphology. The phylogenetic signalling in both centroid size and canal morphology was statistically significant (*p* = 0.007, and *p* = 0.001, respectively), suggesting that closely related species tend to have similarly sized labyrinths and similar canal morphologies.

Both specimens of *Champsosaurus* plot close to the non-avian archosauromorphs and *Youngina* (see Discussion for phylogenetic implications). Within the non-avian archosauromorph group, the *Champsosaurus* specimens plot closest to the aquatic taxa (Fig. 10; e.g., crocodilians and turtles), suggesting that the semicircular canals of *Champsosaurus* are most similar in morphology to aquatic archosauromorphs.

ANCOVA (see Supplementary Table S2) indicates a moderate and significant relationship between ecology and canal shape (R^2^ = 0.25979, *p* = 0.0004), and a weak but significant relationship between centroid size and canal shape (R^2^ = 0.04334, *p* = 0.0045). The interaction between ecology and centroid size was found to have a weak and insignificant relationship with canal shape (R^2^ = 0.02470, *p* = 0.4539).

PGLS (see Supplementary Table S3) indicates similar relationships to the ANCOVA, with a moderate relationship between ecology and canal shape that approaches significance (R^2^ = 0.21451, *p* = 0.0173; Bonferroni corrected α-level = 0.0166), and a weak, but significant relationship between centroid size and canal shape (R^2^ = 0.02882, *p* = 0.0065). The interaction between ecology and centroid size was also found to have a moderate and significant relationship with ecology (R^2^ = 0.09681, *p* = 0.0026). It is interesting to note that the correlation coefficients for ecology and centroid size were lower in the PGLS than in the ANCOVA, and the correlation coefficient for the interaction of ecology and centroid size was higher in the PGLS than in the ANCOVA. This is most likely because there is an interaction between ecology, centroid size, and phylogeny, meaning that ecology and centroid size are not totally independent of phylogeny^13^. This is supported by Bloomberg’s K value for centroid size (K = 0.1793, *p* = 0.007), suggesting that centroid size is significantly influenced by phylogeny.

**Figure 12:**
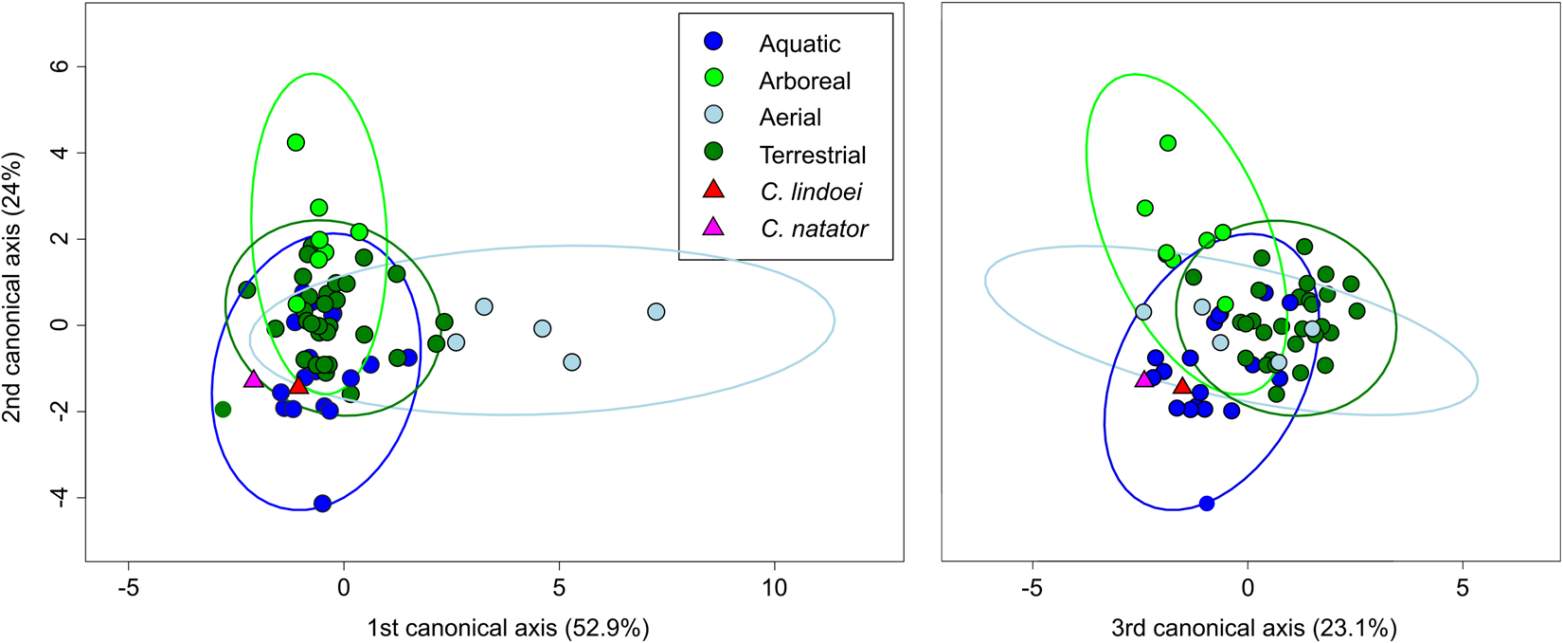
CV1 vs CV2 (left) representing 76.9% of the total between-group variation, and CV3 vs CV2 (right) representing 47.1% of the total between-group cariation. 95% confidence ellipses of each ecological group are plotted.

Visualization of the CVA (Fig. 12) and posterior probabilities (see Supplementary Table S4) show that the ecological groups occupy significantly different regions of morphospace (p < 0.01). The classification accuracy of the CVA was approximately 85%. CV1 is mostly separated by PC1, where positive CV1 values represent increased curvature of the anterior canal, an anterodorsally angled posterior canal, and an anteroventrally angled lateral canal with greater curvature anteriorly. Aerial taxa plot towards CV1 positive, while arboreal, aquatic, and terrestrial taxa plot towards CV1 negative. Both *Champsosaurus* specimens plot towards CV1 negative (Fig. 12). CV2 is mostly influenced by PC2, where positive CV2 values represent anteroposterior elongation and lateral torion of the anterior canal, a more widely curved posterior canal, and greater torsion of the lateral canal. Arboreal taxa plot towards CV2 positive, while aquatic, terrestrial, and aerial taxa plot towards CV2 negative. *Champsosaurus lindoei* and *C. natator* plot towards CV2 negative (Fig. 12). CV3 is mostly influenced by PC3, where negative CV3 values represent a smaller, more eliptical anterior canal, a more acute angle between the anterior and posterior canals, and a more widely curved lateral canal (Fig. 12). Terrestrial taxa plot towards CV3 positive, and aquatic, arboreal, and aerial taxa plot towards CV3 negative. Both *Champsosaurus* species plot towards CV3 negative.

Posterior probabilities of Mahalanobis distances found that *C. natator* occupies a significantly different region of morphospace from the arboreal, aerial, and terrestrial ecological groups (*p* < 0.0001), but does not occupy a significantly different region from the aquatic group (see Supplementary Table S5). Similarly, *C. lindoei* occupies a significantly different region of morphospace from the arboreal and aerial groups (*p* < 0.0001), but does not occupy a significantly different region of morphospace from the aquatic or terrestrial groups. It should be noted that the difference between *C. lindoei* and the terrestrial group approaches significance (*p* = 0.019, Bonferroni corrected α-level = 0.00625), and is significant in the uncorrected pairwise comparisons. Both *Champsosaurus* specimens plot well within the 95% confidence ellipse for the aquatic group, and log-likelihood estimations clearly assign both *Champsosaurus* specimens to the aquatic group (>0.95 likelihood).

## Discussion

### The brain endocast

Overall, the brain endocasts of CMN 8920 and CMN 8919 are typical of other exinct diapsids, such as some phytosaurs (e.g., *Pseudoplatus*)^55^ and early turtles (e.g., *Proganochelys*)^60^, possessing large olfactory lobes, a large pineal body, and small optic lobes and flocculi. A major difference between the brain endocast of *C. lindoei* and *C. natator* is the variation in brain endocast flexure. *Champsosaurus lindoei* does not exhibit any flexure (Fig. 2), while *C. natator* exhibits strong cerebral and pontine flexures (Fig. 3). The cerebral and pontine flexures of CMN 8919 are both approximatley 22°, resulting in parallel cerebral and medullar axes that is typical of reptiles^61^. Brain endocast flexure is known to vary substantially across taxa (e.g., nearly 0° in *Iguanodon*^62^; approximatley 55° in *Caiman crocodilus*^63^), but there is an overall trend towards greater flexure in smaller animals and less flexure in larger animals^61^. This is due to the negative allometric scaling of the brain, where smaller animals tend to have a proportionatley larger brain that is more restricted by the size limits of the skull and spatial limits from other cranial structures, such as the eyes^63,64^. As a result, smaller animals tend to have increased flexure of the brain endocast to accommodate the spatial restrictions of the skull anteroposteriorly^63^. This trend also holds true through ontogeny, where young animals tend to have greater flexure of the brain endocast that decreases as the animal grows^63^.

*Champsosaurus* violates this trend, where the smaller *C. lindoei* possesses no flexure, and the larger *C. gigas* possesses prominent flexure. This may also be due to spatial constraints in the skull, but is likely due to the dorsoventral compression of the skull, as opposed to anteroposterior constraint from the orbits. This is because the skull of *Champsosaurus* is quite long, but dorsoventrally flat, and the brain is more constricted dorsoventrally than it is anteroposteriorly. Therefore, smaller specimens of *Champsosaurus* likely lack flexure due to the limited space dorsoventrally, but the flexure becomes evident in larger individuals where the dorsoventral spatial limitations of the skull are less restricting. Alternatively, this difference in brain flexure may be due to individual variation, similar to the variation proposed in plesiosaur endocast flexure (Allemand *et al*., 2019). These hypotheses should be explicitly tested in the future by comparing the ratio of braincase length to depth with the degree of brain endocast flexure in multiple specimens of *C. lindoei* and *C. natator*.

A notable feature in the braincase of *Champsosaurus* is the dorsal keel on the midline of the parasphenoid (Fig. 5; k). Fox^25^ suggested that this region of the endocast was occupied by the fourth ventricle of the brain, but this is unlikely, given that the ventricles of the brain sit deep within the nervous tissue and do not press against bone^65^. The neurological structure that comprises the ventral portion of the brain stem is the medulla oblongata^65^, and the dorsal keel may indicate the position of the medullary fissure that seperated the right and left sides of the medulla. A small dorsal keel is observed in other reptiles (e.g., *Ctenosaura*^66^; *Euparkeria*^67^), but the prominance of the keel in *Champsosaurus* suggests that the medulla was likely well developed. It is also possible that the dorsal keel represents a suture surface with a posterior cartilagenous extension of the overlying basisphenoid (Fig. 5), which did not fossilize, similar to the cartilagenous basisphenoid observed in lissamphibians^68^.

### Olfaction

Evaluation of olfactory capabilities in extinct taxa relies on measurements of the olfactory bulb^69^, the region of the brain that detects and processes scent information. The walls and floor of the olfactory bulbs did not ossify in *Champsosaurus* and the size of these structures is therefore unknown. Olfactory capabilities in *Champsosaurus*, therefore, cannot be commented upon quantitatively, but basic comparisons can still be made. The olfactory stalks (comprised of the anterior olfactory bulbs that process olfactory information, and the posterior olfactory peduncles that transmitted sensory information from the olfactory bulbs to the cerebrum^69,70^) of *Champsosaurus* comprise half of the length of the entire brain endocast, suggesting that olfaction may have been a powerful sense for this animal.

Erickson^26^ noted that *Champsosaurus* may have possessed a well-developed sense of smell due to the pronounced olfactory chambers of the nasal passages, a conclusion supported here by the prominent olfactory chambers and olfactory duct of CMN 8920 (Fig. 8) that appear comparable in proportional size (relative to the total length of the skull and brain, respectively) to those of modern crocodiles^4^ and gharials^71^. He also stated that a well-developed sense of smell in *Champsosaurus* would be inconsistent with its aquatic habits, because detecting airborne odours would have had little importance for detecting food, but Erickson^26^ did not consider other advantages of high olfactory acuity. Modern crocodilians have a well-developed sense of smell that is used for detecting airborne odours for locating food^72^ and mates^73^. Although *Champsosaurus* was likely piscivorous^26^ and detecting airborne odours would not have provided an advantage for locating aquatic prey, *Champsosaurus* may still have used a developed sense of smell for identifying other nearby animals. It is also possible that the developed sense of smell of *Champsosaurus* allowed it to hunt terrestrial prey in nearshore environments. If so, this hunting behaviour must have been restricted to small terrestrial prey items because the gracile skull of *Champsosaurus* was not suited for acquiring large bodied animals^74^.

Lu *et al*.^27^. suggested that the neochoristodere *Ikechosaurus* may have possessed turbinates to better facilitate olfaction or thermoregulation, but there is no evidence of turbinates in the nasal passage of the *Champsosaurus* specimens examined here. It is still possible that *Champsosaurus* possessed nasal turbinates, or turbinate-like structures, but these would have been cartilaginous because they left no osteological correlate on the walls of the nasal passage.

### Vision

There is no evidence for prominent optic lobes in the *Champsosaurus* endocast, as is consistent with other extinct diapsids (e.g., some phytosaurs^55^ and early turtles^60^), suggesting that *Champsosaurus* had average visual acuity for a stem-group diapsid at best. Some effort has been made to estimate diel activity and colour perception in extinct taxa^75^, but these estimates rely on dimensions of the scleral ring, which *Champsosaurus* lacked. Erickson^26^ suggested that *Champsosaurus* possessed good binocular vision based on the raised position of the orbits on the dorsal surface of the skull and their close spacing, but estimates of the degree of overlap between the visual fields of the two eyes is hindered because there is no way to determine the exact size, position, and orientation of the eyes within the orbits.

### Hearing

Based on the length of the pars inferior, the estimated best hearing frequency of *Champsosaurus* is 1798.8 Hz, and the best hearing range is 2936.5 Hz (overall best hearing range: 330.6–3267.1 Hz). Among the extant taxa examined by Walsh *et al*. (2009), this is most comparable to the American crocodile (*Crocodylus acutus*; best hearing frequency: 1650 Hz; best hearing range: 2700 Hz; overall best hearing range: 300–3000 Hz) and the Indian spiny-tailed lizard (*Uromastyx hardwickii*; best hearing frequency: 1650 Hz; best hearing range: 2700 Hz; overall best hearing range: 300–3000 Hz). It should be noted that the estimated hearing capabilities of *Champsosaurus* are higher than those of most other aquatic reptiles included in this dataset, such as the spectacled caiman (*Caiman crocodylus*; best hearing frequency: 1150 Hz; best hearing range: 1700 Hz: overall best hearing range: 300–2000 Hz), American alligator (*Alligator mississippiensis*; best hearing frequency: 550 Hz; best hearing range: 900 Hz; overall best hearing range: 100–1000 Hz), common snapping turtle (*Chelydra serpentina*; best hearing frequency: 600 Hz; best hearing range: 800 Hz; overall best hearing range: 200–1000 Hz), and the green sea turtle (*Chelonia mydas*; best hearing frequency: 325 Hz; best hearing range: 350 Hz; overall best hearing range: 150–500 Hz). This may be because the pars inferior of tetrapods also contains soft tissue structures around the cochlea, and the length of the pars inferior is therefore an overestimate of the length of the cochlea. Accounting for this overestimation could bring the estimated hearing capabilities of *Champsosaurus* closer to that of most modern crocodiles and turtles; however, no such correction factor has been established.

A striking feature of the endosseous labyrinth of *Champsosaurus* is the absence of a distinct cochlear duct, and the large size of the bulbous pars inferior. Some extinct reptiles did not ossify the anterior, medial, and posterior walls of the pars inferior, and reconstruction of the cochlear duct in these taxa is based solely on the extent of the lagenar crest on the posterior wall of the capsule^67^. This is not the case in *Champsosaurus*, where the anterior and posterior walls of the pars inferior are ossified, and the ventral margin of the pars inferior is clearly indicated by the ventrally oriented fenestra ovalis. Supporting the conclusion that the endosseous labyrinth represents the full extent of the pars inferior in *Champsosaurus* is the fact that the skull does not extend ventral to the fenestra ovalis, so there is no space within the skull in which the pars inferior could have continued ventrally. A large pars inferior is also observed in other aquatic animals (e.g., fish^76^) due to the effectiveness of the sacculus at detecting water-borne sounds via vibration of the saccular otolith. Sea turtles use a combination of both the cochlea and saccular otolith to enable sound detection in water and air. This is facilitated by stapedosaccular strands, unique to turtles, that connect the stapes and fenestra ovalis to the sacculus^77^. It is thought that the stapedosaccular strands facilitate the transmission of vibrations between the sacculus and cochlea for better aquatic sound detection^78^, but the performance of these strands is poorly understood. Interestingly, sea turtles also posses an enlarged sacculus and reduced cochlear duct relative to other reptiles, but it is not known whether the sacculus tends to be larger in sea turtles than in tortoises. Freshwater turtles also possess a large, spherical sacculus that is best adapted for detecting low frequency vibrations (300–500 Hz) underwater, although the tympanum and cochlea still play a major role in sound detection^79^.

Given the inferred highly aquatic lifestyle of *Champsosaurus*, the question arises as to whether they relied solely on sound detection via the sacculus in water, or if they also possessed a tympanum to increase sensitivity to airborne sounds. In all tympanic amniotes, there is a distinct otic notch (conch in lepidosaurs^54^) in the posterior margin of the skull that correlates with the location of the tympanum on the lateral surface of the skull^80,81^. In crown-group diapsids, such as lepidosaurs, archosaurs, and turtles, the otic notch is formed by the posterior margin of the quadrate, but in parareptiles, the otic notch is formed by the posterior margin of the squamosal and quadratojugal^81^. The quadrate of *Champsosaurus* is broad and dorsoventrally flat, forming the floor of the temporal region, but is not a component of the lateral surface of the skull. Instead, the quadratojugal and squamosal comprise the majority of the lateral surface of the skull. Although the posteroventral margin of these elements is slightly concave when viewed laterally, the concavity does not approach the prominent otic notch used to infer the presence of a tympanum in extinct lineages^81–83^. The absence of a prominent otic notch suggests that *Champsosaurus* lacked a tympanum, possibly a retention of the basal amniote condition^84^.

Supporting the hypothesis that *Champsosaurus* were atympanic is the inferred structural role of the stapes in *Champsosaurus*, where the stapes (likely homologous with the choristoderan neomorphic bone^56^) is well sutured to the surrounding bones (primarily the quadrate, prootic, and opisthotic) and could not have articulated with a tympanum. In modern atympanic taxa, such as *Sphenodon* and Serpentes, the stapes articulates with the quadrate, and sound detection is limited to low frequency vibrations^85,86^ that are better able to be conducted through the skull and transmit to the inner ear^86^. This has led researchers to suggest that extinct atympanic reptiles likely would have had similar low frequency hearing capabilities to *Sphenodon* (e.g., *Youngina*^84^). In *Champsosaurus*, the absence of a tympanum, the structural role of the stapes, and the reduction of the cochlea, suggest that the inner ear would have been ineffective at detecting airborne sounds, although they still may have been able to detect some low frequency airborne sounds through vibrations of the skull.

Given the inferred highly aquatic lifestyle of *Champsosaurus*, it is probable that they did not have a need for detecting airborne sounds, and so possessed a large sacculus to better detect waterborne vibrations, similar to the inner ear morphology of modern sea turtles, although the latter are tympanic^78,79^. The sacculus is best able to detect low frequency vibrations, and as a result, animals that use the sacculus to detect sound information, such as fish, are only able to detect low frequency sounds, and usually have a narrow range of hearing^76^. This also holds true for turtles, which have an enlarged sacculus and reduced cochlea, and are only able to detect low frequency sounds^7^. It therefore stands to reason that the estimated hearing capability of *Champsosaurus* based on the equations derived from Walsh *et al*.^7^ is overestimated, and these animals would have been adapted to detecting low frequency water borne vibrations, similar to modern sea turtles. A large sacculus and short cochlear duct are also apparent in the endosseous labyrinth of pleisiosaurs^14^, similar to sea turtles and *Champsosaurus*. This suggests that a short cochlea and large sacculus are typical for an aquatic reptile, and that the inner ear of *Champsosaurus* is sufficiently adapted for hearing in an aquatic environment.

An interesting similarity also exists between the inner ear of *Champsosaurus* and many fossorial tetrapods. An enlarged sacculus in fossorial taxa better facilitates the detection of substrate vibrations^12,87,88^. This seems unusual for *Champsosaurus*, given the inferred highly aquatic lifestyle of these animals, but the large sacculus of *Champsosaurus* may have also facilitated the detection of substrate vibrations. It is therefore possible that *Champsosaurus* spent a great deal of time at the bottom of slow-moving bodies of water, such as rivers and lakes, and detected sound vibrations via the substrate, contrary to the vertically floating reconstruction proposed by Erickson^26^. The ventral orientation of the fenestra ovalis may have increased the sensitivity of the sacculus to vibrations received ventrally from the substrate, similar to some modern urodeles^89–91^.

### Phylogeny and the endosseous labyrinth

Bloomberg *et al*.^51^ determined that most traits other than body size tend to show less phylogenetic signal than expected under Brownian motion (K < 1), an observation that is supported here by the low K value associated with canal morphology. Despite the low K value, the data presented in Fig. 10, Supplementary Fig. S5, and Supplementary Table S1 demonstrate that there is a strong phylogenetic signal in the morphology of neodiapsid semicircular canals. Three distinct groups are apparent when plotting PC1 vs PC2: Aves, Lepidosauria, and non-avian archosauromorphs (Fig. 10). Both *Champsosaurus* specimens plot among the non-avian archosauromorphs, supporting some phylogenetic assessments that place Choristodera as a stem-group of early archosauromorphs^92,93^. However, *Youngina* also plots among the non-avian archosauromorphs, suggesting that archosauromorphs and *Champsosaurus* retained the plesiomorphic neodiapsid morphology of the semicircular canals. It is therefore equally parsimonious to suggest that *Champsosaurus* (and choristoderes) are basal to crown diapsids, and our data at present are unable to distinguish between the two hypotheses. Although these data are consistent with the hypothesis that choristoderes are basal archosauromorphs, alternative relationships are underdetermined by the available evidence due to the absence of basal lepidosauromorph labyrinths in our dataset.

Ideally, a larger sample size would be used in the PCA to better represent some modern and extinct phylogenetic groups, but this is hindered by relatively low variation in some extant groups (e.g., modern archosaurs are solely Aves and Crocodylia), and the limited abundance of fossil CT data. Despite this, the data presented here provide novel evidence suggesting that choristoderes are either basal archosauromorphs or basal to crown-diapsids, but their exact phylogenetic position remains uncertain. The morphology of the inner ear should therefore be used as a character in future phylogenies to evaluate the systematics of Choristodera, but is beyond the scope of this study. Additionally, turtles plot among the non-avian archosauromorphs, supporting a growing body of morphological^34^ and molecular^22^ evidence for an archosauromorph origin of Pantestudines^36^. Similar to choristoderes, however, this may also suggest that turtles are basal to Archosauromorpha, and our data are unable to distinguish between these two hypotheses.

Significant phylogenetic signal (*p* = 0.007) was also observed in centroid size, but was less than expected under Brownian motion (K = 0.1793), consistent with previous studies that have found significant phylogenetic signal in centroid size^13^. This phenomenon is possibly due to the significant correlation between centroid size, ecology, and canal shape (see Supplementary Tables S2 and S3). Several studies have found that semicircular canals with a larger radius of curvature, and therefore a greater centroid size^8^, tend to be more sensitive to angular movement^8,94^. Agile species therefore tend to have canals with a greater radius of curvature than sedentary species^95,96^. Both ecology and canal shape are also known to be strongly influenced by phylogeny^10,13^, and the close relationship of centroid size with ecology and canal shape makes the strength of phylogenetic signaling unsurprising. Although beyond the scope of this study, future analyses should compare centroid sizes between distinct ecological groups to describe possible patterns in the variation of centroid size.

### Ecology and the endosseous labyrinth

It has repeatedly been suggested that canal morphology is closely associated with ecology^9–11,14,15^, but as discussed above, the morphology of the canals also carries a strong phylogenetic signal^13^. Shape analyses have repeatedly been conducted on reptiles, but have focused on relatively closely related groups, such as snakes^12^ or squamates as a whole^9^. Previous studies have also demonstrated that semicircular canal morphology may converge between separate lineages when they have similar ecologies, but within reptiles, these studies have also focused on relatively closely related taxa (e.g., anolis lizards^13^; Serpentes^15^) and have not investigated the influence of ecology on larger evolutionary scales. The data presented here suggest that, despite the strength of phylogenetic signaling on such large scales, the morphology of the semicircular canals is additionally influenced by ecology (see Supplementary Tables S2 and S3). When evolutionary lineages were considered independent (ANCOVA), the correlation between ecology was found to be moderate and significant (see Supplementary Table S3). When lineages were considered dependant (PGLS), the correlation was found to moderate but statistically insignificant (see Supplementary Table S3). The difference in significance between ANCOVA and PGLS is likely because phylogenetic signalling is responsible for far greater variation than ecology, but the moderate size of the correlation coefficient for ecology in PGLS (0.21451) suggests that there is still a biologically relevant correlation between the two factors, even over a large evolutionary scale.

Some studies^97,98^ have suggested that the orthogonality of mammal semicircular canals is more closely associated with canal sensitivity than canal shape, where species that move dynamically tend to have canals oriented closer to 90 degrees from one another than species with less dynamic movements. Along the PCAs, the angle of the anterior and posterior canals relative to one another is not a major component of shape variation, (e.g., PC3, representing 6.80% of the variation), suggesting that orthogonality is not a major component of the morphological variation in neodiapsid semicircular canals. In the future, canal angle could be tested separately by measuring the angles between the semicircular canals and testing for differences via PGLS, following the methods of Malinzak *et al*.^97^.

Within the PCA, both *Champsosaurus* species plot among the aquatic non-avian archosauromorphs, suggesting that the semicircular canal morphology of *Champsosaurus* was adapted to an aquatic lifestyle; a canal morphology characterized by shorter, less curved canals, less torsion of the posterior canal, and less torsion of the lateral canal. This conclusion is further corroborated by the CVA, which plots both *Champsosaurus* species closest to the aquatic group.

A notable difference in morphology between the labyrinths of *C. lindoei* and *C. natator* is the angle of the lateral semicircular canal relative to the long axis of the skull (Fig. 7). The lateral canal of *C. lindoei* is angled approximately −15.8° to the long axis of the skull, and the lateral canal of *C. natator* is angled approximately 13.3° to the long axis of the skull, a difference of approximately 29°. Previously, the angle of the lateral canal relative to the long axis of the skull has been used to reconstruct head posture in extinct taxa^99–101^, but some evidence suggests that the angle of the lateral canal is highly variable and does not accurately reflect head posture in some groups of animals (e.g., saurischians^16^). The variation in the lateral canal of *Champsosaurus* supports the notion that the angle of the lateral canal can be highly variable, as *Champsosaurus* species are thought to have had similar ecologies and behaviour to one another^18^ and are therefore unlikely to have had significantly different habitual head postures.

Although the relationship between semicircular canal morphology and ontogeny has received some attention^102,103^, there is not enough information here to comment on whether the variation in canal morphology between CMN 8920 and CMN 8919 is ontogenetic, due to small sample size. Slow moving taxa, such as sloths, have greater variation in canal morphology than most animals due to their slow-moving lifestyle that has lessened selective pressures on the morphology of the semicircular canals^10^. It is possible that the variation in canal shape in *Champsosaurus* is due to a sedentary lifestyle, but a much larger sample size is needed to determine if the variation in *Champsosaurus* canal morphology is atypical. A study describing the semicircular canals of several individuals at varying ontogenetic stages within each species of *Champsosaurus* would also describe whether the variation in morphology between CMN 8920 and CMN 8919 is typical for the genus, or is due to interspecific or ontogenetic variation between^104^.

Posterior probabilities of Mahalanobis distances demonstrate that the *Champsosaurus* species exhibit no significant differences from the aquatic group (see Supplementary Table S5), although *C. lindoei* also does not occupy a significantly different region of morphospace from the terrestrial group. However, log-likelihood estimates strongly support the inclusion of both *Champsosaurus* specimens within the aquatic group (see Supplementary Table S5). This supports previous notions based on skeletal and sedimentological evidence^26^ that *Champsosaurus* was adapted for an aquatic lifestyle, and suggests that the sensory anatomy of *Champsosaurus* was similar to that of other aquatic reptiles.

## Conclusions

Detailed analysis of the braincase of two specimens of *Champsosaurus* revealed that it was poorly ossified anteriorly, but well ossified posteriorly, similar to other diapsids. The morphology of the brain endocast is similar to that of basal archosauromorphs, possessing an enlarged pineal body and olfactory bulbs, and reduced optic lobes and flocculi. Although the olfactory ability of *Champsosaurus* cannot be estimated quantitatively due to lack of ossification, the olfactory stalks of the brain endocast and olfactory chambers of the nasal passages are quite large, and likely facilitated good olfaction. There is no evidence of turbinates in the nasal passage, and if *Champsosaurus* did possess these structures, they were likely entirely cartilaginous and left no osteological correlates. The small size of the optic lobes and flocculi suggest that *Champsosaurus* had, at best, average sight for a diapsid reptile. Based on the length of the pars inferior, the hearing capabilities of *Champsosaurus* were typical for a reptile, but this is likely an overestimate of the hearing capabilities due to a lack of constraint on cochlear length presented in the endosseous labyrinth reconstruction. An expansion of the sacculus within the pars inferior of *Champsosaurus* is interpreted as conferring sensitivity to low frequency sounds and vibrations, similar to modern turtles. Posterior probabilities and log-likelihood estimates demonstrate that the morphology of the semicircular canals of *Champsosaurus* are most similar to other aquatic reptiles, suggesting that the semicircular canals of *Champsosaurus* were adapted for an aquatic lifestyle.

To our knowledge, this is the first study to analyse the morphology of semicircular canals across Neodiapsida. The PCA suggests that birds, non-avian archosauromorphs, and lepidosaurs possess significantly different canal morphologies due to high phylogenetic signalling in the morphology of the semicircular canals. The *Champsosaurus* species and turtles plot among the non-avian archosauromorphs, but it is not currently possible to determine if choristoderes are basal archosauromorphs or basal to crown-diapsids. The data presented here are consistent with the hypothesis that choristoderes are basal archosauromorphs, but the relationship of choristoderes to lepidosauromorphs remains uncertain until labyrinths from more basal members become available.

## Supplementary information

Supplementary Information.
