# Supplementary material for: The internal cranial anatomy of Champsosaurus (Choristodera: Champsosauridae): Implications for neurosensory function
Source: Sci Rep. 2020 Apr 28;10:7122. doi: 10.1038/s41598-020-63956-y (PMC7188685; doi:10.1038/s41598-020-63956-y)
Supplement: Supplementary file 1 — Supplementary Information. [file 41598_2020_63956_MOESM1_ESM.pdf]

The internal cranial anatomy of *Champsosaurus* (Choristodera: Champsosauridae): Implications for neurosensory function

Thomas W. Dudgeon[1*], Hillary C. Maddin[1], David C. Evans[2,3], Jordan C. Mallon[1,4]

1 Department of Earth Sciences, Carleton University, Ottawa, Canada;
2 Vertebrate Palaeontology, Royal Ontario Museum, Toronto, Canada;
3 Department of Ecology and Evolutionary Biology, University of Toronto, Toronto, Canada;
4 Beaty Centre for Species Discovery and Palaeobiology Section, Canadian Museum of Nature, Ottawa, Canada.

* Corresponding author thomasdudgeon@cmail.carleton.ca

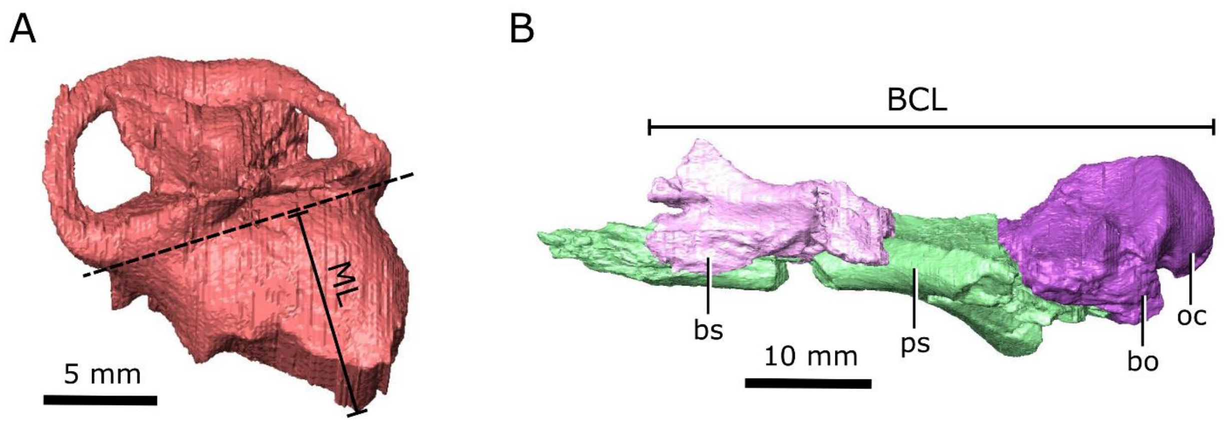

Figure S1: Measurements used for the estimation of auditory capabilities. **A** maximum endocochlear duct length (ML); **B** basicranial length (BCL). *Abbreviations: bo, basioccipital; bs, basisphenoid; oc, occipital condyle; ps, parasphenoid.* Images generated in Amira 5.4.3 (https://www.fei.com/software/amira/) and processed in Inkscape 0.92 (https://inkscape.org/).

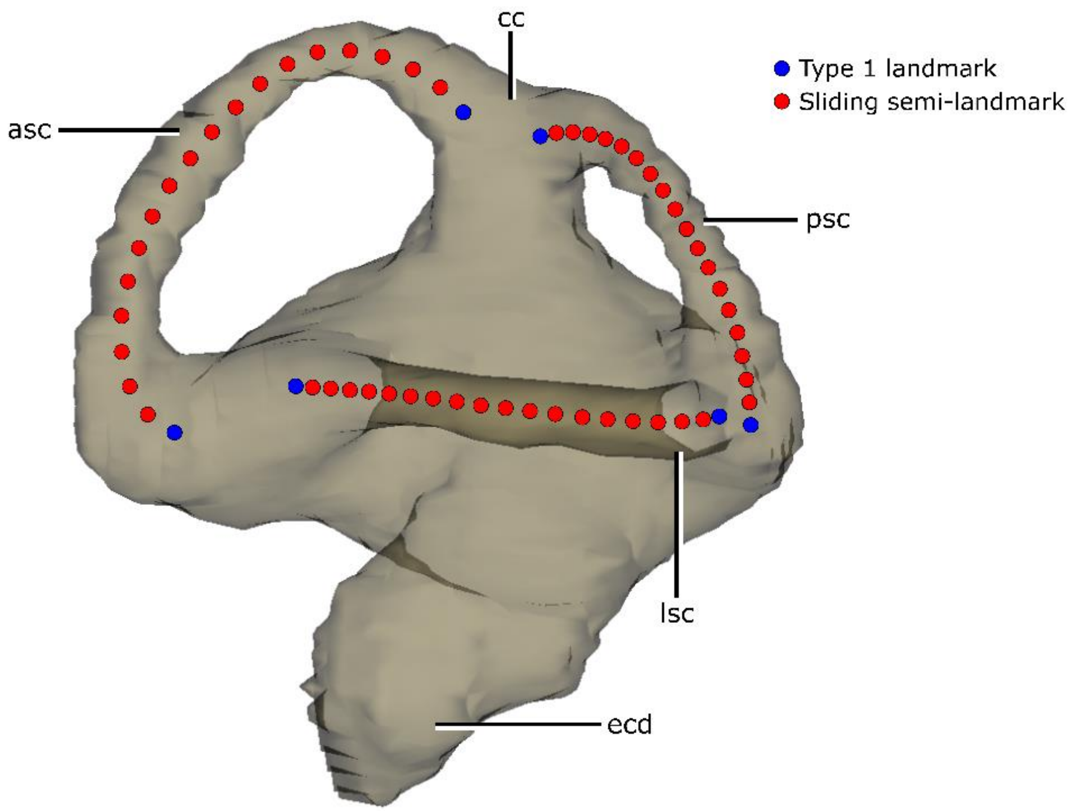

Figure S2: Landmark locations for the three semicircular canals of *Tomistoma schegelii*. *Abbreviations: asc, anterior semicircular canal; cc, crus communis; ecd, endocochlear duct; lsc, lateral semicircular canal; psc, posterior semicircular canal.* Image generated in MorphoDig 1.4.0 (https://morphomuseum.com/morphodig) and processed in Inkscape 0.92 (https://inkscape.org/).

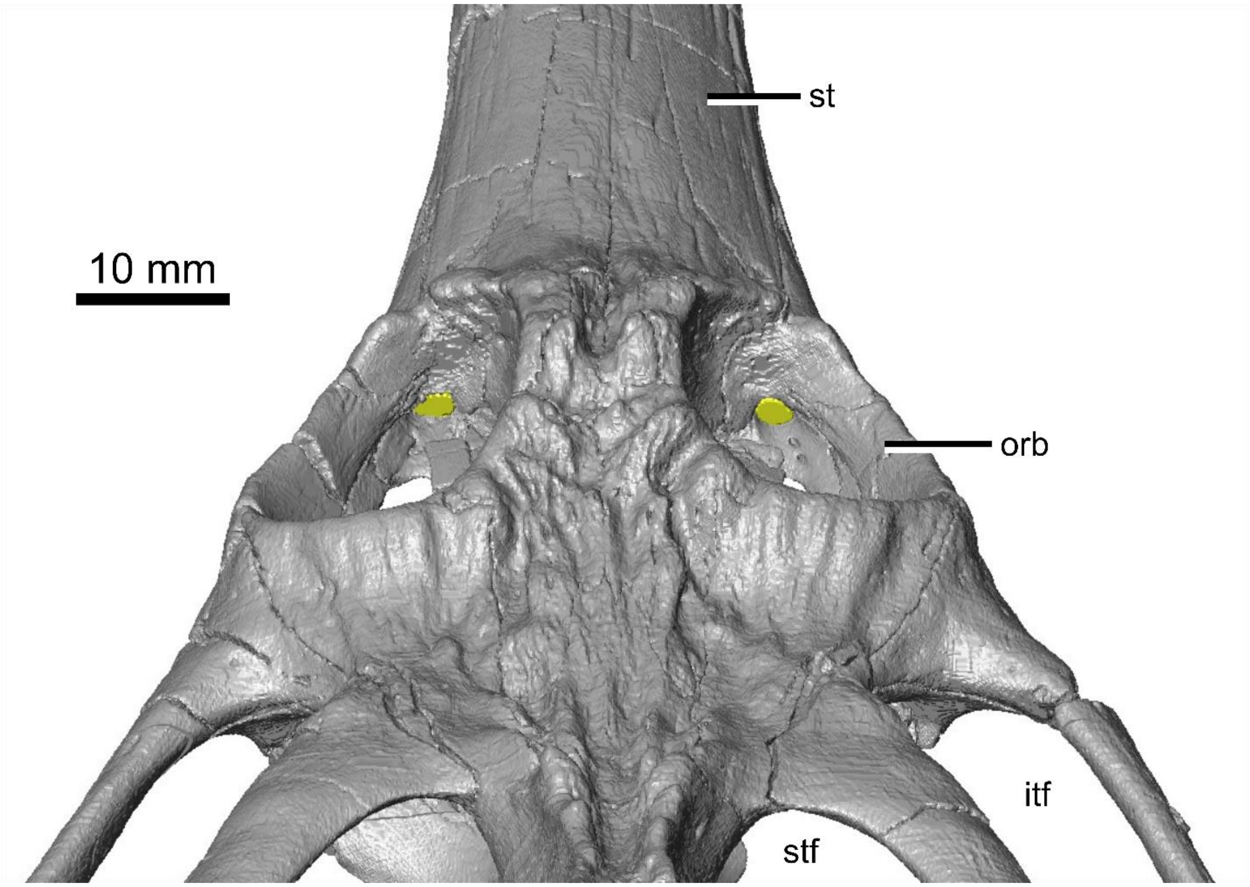

Figure S3: Posterodorsal view of the orbital region of the digitized skull of *Champsosaurus lindoei* (CMN 8920). The canal for the maxillary branch of the trigeminal nerve is visualized in yellow. *Abbreviations: itf, infratemporal fenestra; orb, orbit; st, snout; stf, supratemporal fenestra*. Image generated in Amira 5.4.3 (https://www.fei.com/software/amira/).

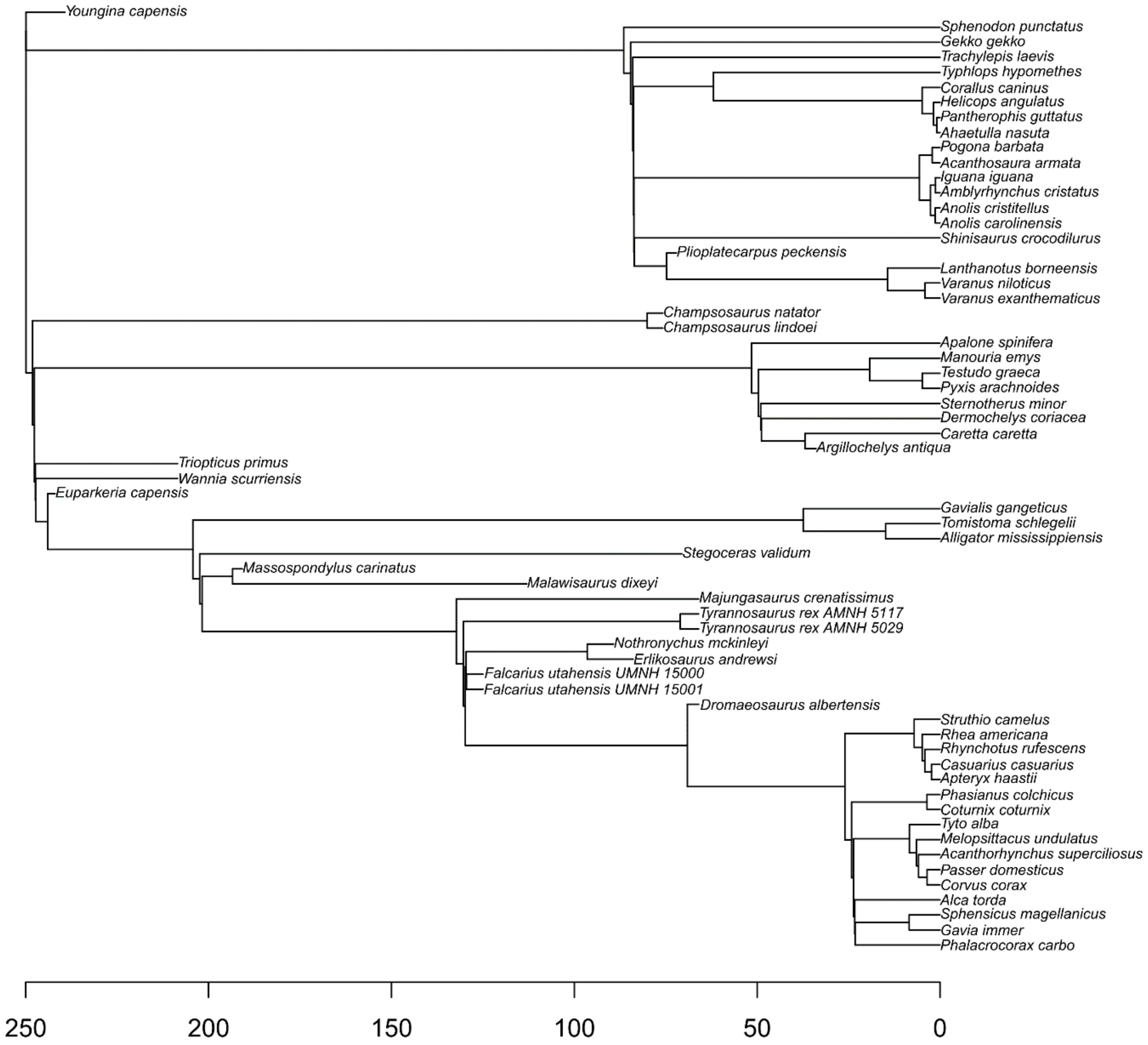

Figure S4: Consensus time-calibrated phylogeny of taxa used in the PCA. Scale bar indicates millions of years before present. Image generated in RStudio 1.2.5019 (https://rstudio.com/).

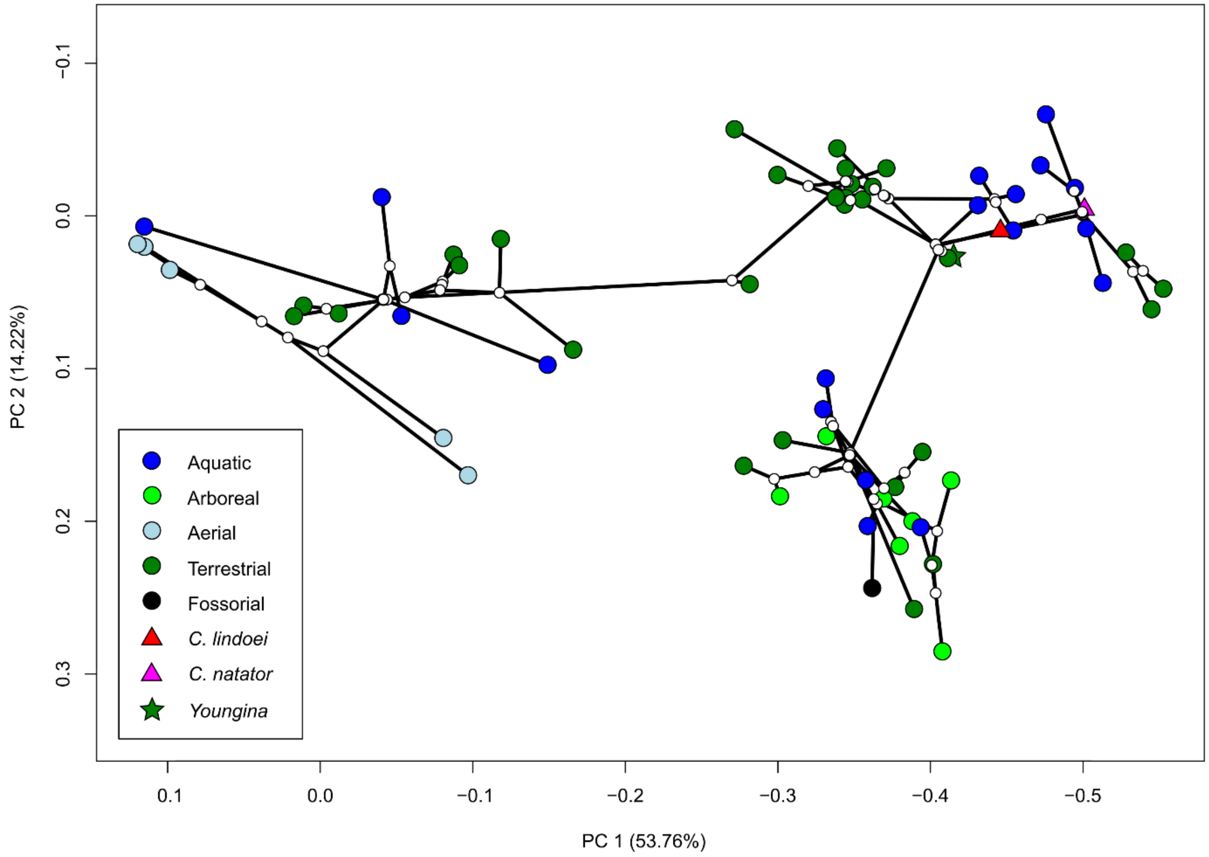

Figure S5: Consensus time-calibrated phylogeny (Supplementary Fig. S4) of included taxa projected into the morphospace of PC1 and PC2. Taxa are colour coded based on ecology. It should be noted that projecting the phylogeny onto PC1 vs PC2 resulted in the PC scores being flipped along PC2; however, this PCA was created solely for visualization of the phylogeny in morphospace and the resulting PC scores were not used in the subsequent analyses. Therefore, the flipping of the PC scores along PC2 in Supplementary Fig. S4 does not influence the results of this study.

Table S1: Bloomberg's K value and associated p-value for both centroid size and landmark coordinates of the endosseous labyrinths. Bloomberg's multivariate K statistic calculated using 1000 permutations. Bold indicates significance (alpha = 0.025).

	K_{mult} statistic	p-value
Centroid Size	0.1793	**0.007**
Coordinates (shape)	0.1496	**0.001**

Table S2: ANCOVA of ecology, centroid size, and the interaction between ecology and centroid size against canal shape. Bold indicates significance (α = 0.0166). *Abbreviations: d.f., degrees of freedom; F, F statistic; MS; mean square;* R^2*, coefficient of determination; SS, sum of squares; Z, Z score.*

	d.f.	SS	MS	R^2	F	Z	p-value
Ecology	4	0.9649	0.241217	0.25979	5.2177	4.3913	**0.0004**
Centroid	1	0.1610	0.160951	0.04334	3.4815	2.8402	**0.0045**
Ecology: Centroid	3	0.0918	0.030585	0.02470	0.6616	0.0787	0.4539
Residuals	54	2.4965	0.046231	0.67217			
Total	62	3.7140					

Table S3: Phylogenetic generalized least squares of semicircular canal shape against ecology, and semicircular canal centroid size. Bold indicates significance (α = 0.0166). *Abbreviations: d.f., degrees of freedom; F, F statistic; MS; mean square;* R^2*, coefficient of determination; SS, sum of squares; Z, Z score.*

	d.f.	SS	MS	R^2	F	Z	p-value
Ecology	4	0.034176	0.0085440	0.21451	4.3885	2.8928	0.0173
Centroid	1	0.004592	0.0045921	0.02882	2.3587	2.4911	**0.0065**
Ecology: Centroid	3	0.015424	0.0051413	0.09681	2.6408	3.0154	**0.0026**
Residuals	54	0.105132	0.0019469	0.65986			
Total	62	0.159324					

Table S4: Posterior probabilities (10 000 permutations) of significant differences between the ecological groups based on Mahalanobis distances. Bold indicates significance ($\alpha = 0.0083$).

	Aquatic	Aerial	Arboreal	Terrestrial
Aquatic	-	**0.0001**	**0.0009**	**0.0002**
Aerial	**0.0001**	-	**0.0001**	**0.0001**
Arboreal	**0.0009**	**0.0001**	-	**0.0017**
Terrestrial	**0.0002**	**0.0001**	**0.0017**	-

Table S5: Posterior probabilities (10 000 permutations) and log-likelihood estimations of *Champsosaurus* species belonging to separate ecological groups. Bold indicates significance (α = 0.00625). Posterior probabilities less than alpha suggest that *Champsosaurus* is significantly different from that ecological group.

	Log-likelihood	Posterior probability			
		Aquatic	Aerial	Arboreal	Terrestrial
Champsosaurus lindoei	Aquatic (0.969)	0.820	**< 0.0001**	**< 0.0001**	0.019
Champsosaurus natator	Aquatic (0.999)	0.199	**< 0.0001**	**< 0.0001**	**< 0.0001**

Table S6: Taxa included in the analysis that were acquired via Morphosource, with their specimen ID, specimen institution, Morphosource media number, and ecological group used in the PCA and CVA.

Group	Species	Specimen	Specimen Institution	Media number	Ecological Group
**Archosauromorpha**	*Apalone spinifera*	FMNH:22178	Field Museum of Natural History	M22038	Aquatic
	Alca torda	NMS:Z 2000.08.105	National Museum of Scotland	M17321	Aquatic
	Acanthorhynchus superciliosus	NHMUK:zoo:s/1966.51.209	Natural History Museum	M17246	Aerial
	Alligator mississippiensis	OUVC:9761	Ohio University	M39878	Aquatic
	Apteryx haastii	NMS:Z:1913.13.748	National Museum of Scotland	M17247	Terrestrial
	Corvus corax	NHMUK:zoo:s/1973.66.160	Natural History Museum	M17389	Aerial
	Coturnix coturnix	NMS:Z.1931.43_Coturnix	National Museum of Scotland	M17723	Terrestrial
	Dermochelys coriacea	UMZC:R:3031	University Museum of Zoology	M22024	Aquatic
	Casuarius casuarius	NHMUK:zoo:1939.12.9.964	Natural History Museum	M17005	Terrestrial
	Gavia immer	NCSM-herp-93545	National Museum of Scotland	M17717	Aquatic
	Gavialis gangeticus	uf:herp:118998	Florida Museum of Natural History	M20938	Aquatic
	Manouria emys	UMMZ:herps:227759	University of Michigan Museum of Zoology	M38591	Terrestrial
	Melopsittacus undulatus	NHMUK:ZOO:s/1966.51.59	Natural History Museum	M17392	Aerial
	Passer domesticus	NMS:Passer_unreg	National Museum of Scotland	M17720	Aerial
	Phalacrocorax carbo	NMS:Z.2000.27.04	National Museum of Scotland	M17423	Aquatic
	Phasianus colchicus	NMS:Z.1931.43_Phasianus	National Museum of Scotland	M17725	Terrestrial
	Pyxis arachnoides	UMMZ:herps:227768	University of Michigan Museum of Zoology	M38593	Terrestrial
	Rhynchotus rufescens	NMS:Z.1890.28.9	National Museums of Scotland	M17408	Terrestrial
	Spheniscus magellanicus	USNM:birds:347603	National Museum of Natural History	M15987	Aquatic
	Stegoceras validum	UALVP-2	University of Alberta Laboratory for Vertebrate Paleontology	M22180	Terrestrial

	Sternotherus minor	FMNH:211696	Field Museum of Natural History	M22123	Aquatic
	Struthio camelus	NHMUK:ZOO:S/1927.2.5.1	Natural History Museum	M17394	Terrestrial
	Testudo graeca	MCZ:herp:r-18161	Museum of Comparative Zoology	M46170	Terrestrial
	Triopticus primus	TMM:31100-1030	University of Texas Vertebrate Paleontology Collections	M7293	Terrestrial
	Tyto alba	NHMUK:ZOO:s/1981.39.1	Natural History Museum	M17396	Aerial
	Wannia scurriensis	TTU P:00539	University of Texas Vertebrate Paleontology Collections	M12518	Aquatic
**Lepidosauria**	*Amblyrhynchus cristatus*	uf-herp-41558	Florida Museum of Natural History	M20753	Aquatic
	Acanthosaura armata	ypm:vz:ypm herr 000729	Yale Peabody Museum	M30001	Arboreal
	Ahaetulla nasuta	UMMZ:herps:225477	University of Michigan Museum of Zoology	M16377	Arboreal
	Anolis carolinensis	NCSM:herp:93545	North Carolina Museum of Natural Sciences	M20838	Arboreal
	Anolis cristitellus	UF:Herp:47686	Florida Museum of Natural History	M12744	Arboreal
	Corallus caninus	UMMZ:herps:176798	University of Michigan Museum of Zoology	M22254	Arboreal
	Gekko gecko	SHSVM:H:0001-2014	Sam Houston State Vertebrate Museum	M13432	Arboreal
	Helicops angulatus	UMMZ:herps:65852	University of Michigan Museum of Zoology	M29152	Aquatic
	Pogona barbata	UMM:herps:132851	University of Michigan Museum of Zoology	M38895	Terrestrial
	Shinisaurus crocodilurus	UF-H-60925	Florida Museum of Natural History	M12414	Aquatic
	Sphenodon punctatus	UF:Herp:11978	Florida Museum of Natural History	M8675	Terrestrial
	Trachylepis laevis	cas:herp:254838	California Academy of Science	M15846	Terrestrial

Table S7: Taxa included in the analysis that were acquired via Digimorph (University of Texas High-Resolution X-ray CT Facility), with their specimen ID, specimen institution, NSF grant number, and ecological group used in the PCA and CVA.

Group	Species	Specimen	Specimen Institution	NSF grant num.	Ecological Group
**Archosauromorpha**	*Tomistoma schlegelli**	TMM M-6342	Texas Memorial Museum	IIS-0208675	Aquatic
	Rhea Americana	TMM M-6721	Texas Memorial Museum	IIS-9874781	Terrestrial
**Lepidosauria**	*Lanthanotus borneensis*	FMNH 148589	Field Museum of Natural History	IIS-0208675 and EF-0334961	Aquatic
	Varanus exanthematicus	FMNH 58299	Field Museum of Natural History	IIS-0208675 and EF-0334961	Terrestrial

*Additional credit to Chris Brochu

Table S8: Taxa included in this analysis that were acquired via personal contact with researchers, with their specimen ID, source, specimen institution, and ecological group used in the PCA and CVA.

Group	Species	Specimen	Source	Specimen Institution	Ecological Group
**Choristodera**	*Champsosaurus lindoei*	CMN FV 8920	This study	Canadian Museum of Nature	?
	Champsosaurus natator	CMN FV 8919	This study	Canadian Museum of Nature	?
**Neodiapsida**	*Youngina capensis*	AMNH 5561	Nicholas Gardner	American Museum of Nature History	Terrestrial
**Archosauromorpha**	*Euparkeria capensis*	SAM-PK-7696	Gabriela Sobral, and the Digital Collection of the Museum für Naturkunde Berlin	South African Museum	Terrestrial
	Argillochelys antiqua	NHMUK:38955	Serjoscha Evers	Natural History Museum	Aquatic
	Caretta caretta	NHMUK:1940.3.15.1	Serjoscha Evers	Natural History Museum	Aquatic
	Dromaeosaurus albertensis	AMNH 5356	Lawrence Witmer	American Museum of Natural History	Terrestrial
	Malawisaurus dixeyi	Mal 202-1	Kate Andrzejewski and Mike Polcyn	Malawi Department of Antiquities	Terrestrial
	Massospondylus carinatus	BP/1/5241	Kimberley Chapelle	University of Witwatersrand	Terrestrial
	Falcarius utahensis	UMNH VP 15000	Stephan Lautenschlager	Natural History Museum of Utah	Terrestrial
	Falcarius utahensis	UMNH VP 15001	Stephan Lautenschlager	Natural History Museum of Utah	Terrestrial
	Erlikosaurus andrewsi	IGM 100/111	Stephan Lautenschlager	Geological Institute of the Mongolian Acadamy of Science	Terrestrial
	Majungasaurus crenatissimus	FMNH-PR-2100	Lawrence Witmer	Field Museum of Natural History	Terrestrial
	Nothronychus mckinleyi	AZMNH-2117	Stephan Lautenschlager	Arizona Museum of Natural History	Terrestrial
	Tyrannosaurus rex	AMNH 5029	Lawrence Witmer	American Museum of Natural History	Terrestrial
	Tyrannosaurus rex	AMNH 5117	Lawrence Witmer	American Museum of Natural History	Terrestrial
**Lepidosauria**	*Plioplatecarpus peckensis*	MOR 1062	Hillary Maddin	Museum of the Rockies	Aquatic
	Iguana iguana	MCZ 2560	Hillary Maddin	Museum of Comparative Zoology	Arboreal
	Pantherophis guttatus	Uncatalogued, available in the Maddin Lab	Fred Gaidies, and Hillary Maddin	Carleton University	Terrestrial

	Typhlops hypomethes	Uncatalogued, available in the Maddin Lab	Hillary Maddin	Carleton University	Terrestrial
	Varanus niloticus	MCZ 1066	Hillary Maddin	Museum of Comparative Zoology	Terrestrial
